# Donor-acceptor substituted phenylethynyltriphenylenes – excited state intramolecular charge transfer, solvatochromic absorption and fluorescence emission

**DOI:** 10.3762/bjoc.6.112

**Published:** 2010-10-18

**Authors:** Ritesh Nandy, Sethuraman Sankararaman

**Affiliations:** 1 Department of Chemistry, Indian Institute of Technology Madras, Chennai 600036, India

**Keywords:** charge transfer, fluorescence, solvatochromism, solvent effect, triphenylene

## Abstract

Several 2-(phenylethynyl)triphenylene derivatives bearing electron donor and acceptor substituents on the phenyl rings have been synthesized. The absorption and fluorescence emission properties of these molecules have been studied in solvents of different polarity. For a given derivative, solvent polarity had minimal effect on the absorption maxima. However, for a given solvent the absorption maxima red shifted with increasing conjugation of the substituent. The fluorescence emission of these derivatives was very sensitive to solvent polarity. In the presence of strongly electron withdrawing (–CN) and strongly electron donating (–NMe_2_) substituents large Stokes shifts (up to 130 nm, 7828 cm^−1^) were observed in DMSO. In the presence of carbonyl substituents (–COMe and –COPh), the largest Stokes shift (140 nm, 8163 cm^−1^) was observed in ethanol. Linear correlation was observed for the Stokes shifts in a Lippert–Mataga plot. Linear correlation of Stokes shift was also observed with *E*_T_(30) scale for protic and aprotic solvents but with different slopes. These results indicate that the fluorescence emission arises from excited state intramolecular charge transfer in these molecules where the triphenylene chromophore acts either as a donor or as an acceptor depending upon the nature of the substituent on the phenyl ring. HOMO–LUMO energy gaps have been estimated from the electrochemical and spectral data for these derivatives. The HOMO and LUMO surfaces were obtained from DFT calculations.

## Introduction

Fluorescent molecular probes that emit in the visible region and whose fluorescence emission is sensitive to environment and solvent polarity are of significant interest due to their versatile applications in chemistry, biology and environmental science [1–4]. Considerable effort has been expended into shifting the fluorescence emission of organic molecules into the visible region. The most commonly employed strategy for the bathochromic shifting of the emission wavelength is to extend the conjugation of the fluorophores with aryl, ethenyl and ethynyl groups. The addition of electron donating and withdrawing substituents to these conjugation enhancing groups also helps in shifting the emission wavelengths further into red region. For example, boron-dipyrrolomethenes (BODIPYs) are a class of molecules whose absorption and fluorescence emission have been fine tuned by suitable substituents [5–7]. Fluorophores emitting in the visible region are important especially in the in vivo study of biological samples. Otherwise the background blue emission of the biological samples interferes with the fluorescence sensing. The mechanism of fluorescence sensing often involves excited state intramolecular charge transfer (ICT) [8–11], photoinduced electron transfer [12–15] and metal ion induced enhancement or quenching of fluorescence [16–19]. Among these, fluorophores that exhibit excited state ICT are very popular. In this context fluorescent donor-π spacer-acceptor (d-π-a) type molecules are of considerable interest and importance. In this class of molecule, the excited state is generally highly polar compared to the ground state due to intramolecular charge transfer from the donor to the acceptor group. The intramolecular charge transfer results in a large dipole moment in the excited state compared to that of the ground state rendering its fluorescence emission sensitive to environment and solvent polarity [20–22].

Pyrene is a prototypical example of a fluorophore and its monomer emission occurs around 380 nm. It has been shifted to as high as 600 nm by multiple substitution by groups that extend the conjugation and also by substituting donor-acceptor groups along the conjugation [23–25]. In addition, pyrene also exhibits excimer emission at a longer wavelength compared to monomer emission which can be used in sensing applications [26–30]. The pyrene chromophore can act as a donor or as an acceptor depending upon the substituent. Pyrene- π spacer-donor and pyrene- π spacer-acceptor type molecules have been widely studied and they have been used in sensing, photo and electro-luminescence applications [31–36]. Unlike pyrene, the triphenylene chromophore has not been widely studied. In contrast to pyrene, triphenylene does not form an excimer in the excited state – emission from the excimer state is very rare for triphenylene chromophore [37–39]. The fluorescence of triphenylene occurs around 348 nm, which is more blue shifted than pyrene monomer emission. Only a few reports on the extension of conjugation of the triphenylene chromophore have appeared. 2,3,6,7,10,11-Hexakis(ethynyl)triphenylene derivatives have been used in photonics as organic light emitting diode (OLED) materials [40–42]. Therefore, it is worthwhile to tune the fluorescence emission of the triphenylene chromophore by the addition of suitable substituents.

**Scheme 1:**
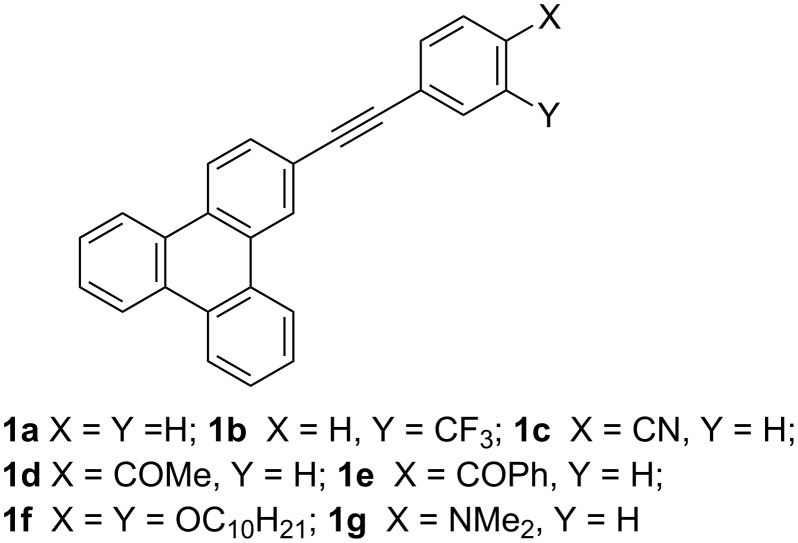
Structures of 2-phenylethynyltriphenylene derivatives.

Herein we report the synthesis of a series of 2-(phenylethynyl)triphenylene derivatives with donor and acceptor substituents on the phenyl ring (Scheme 1). The phenylethynyl group is used to extend the conjugation of the triphenylene chromophore and the substituents on the phenyl ring are used to enhance intramolecular charge transfer (ICT) in the excited state. We have studied the absorption and emission properties, in particular the ICT based solvatochromic fluorescence emission behavior and the correlation of the observed large Stokes shifts with orientation polarizibility (Δ*f*) and solvent polarity (*E*_T_(30) scale). HOMO and LUMO surfaces of these derivatives, obtained from DFT calculations, help in identifying the triphenylene chromophore acting as an acceptor when substituted with electron donating groups and as a donor when substituted with electron withdrawing groups on the phenyl ring. HOMO–LUMO energy gaps have been estimated based on electrochemical studies and compared with those obtained from absorption spectroscopy.

## Results and Discussion

### Synthesis

**Scheme 2:**
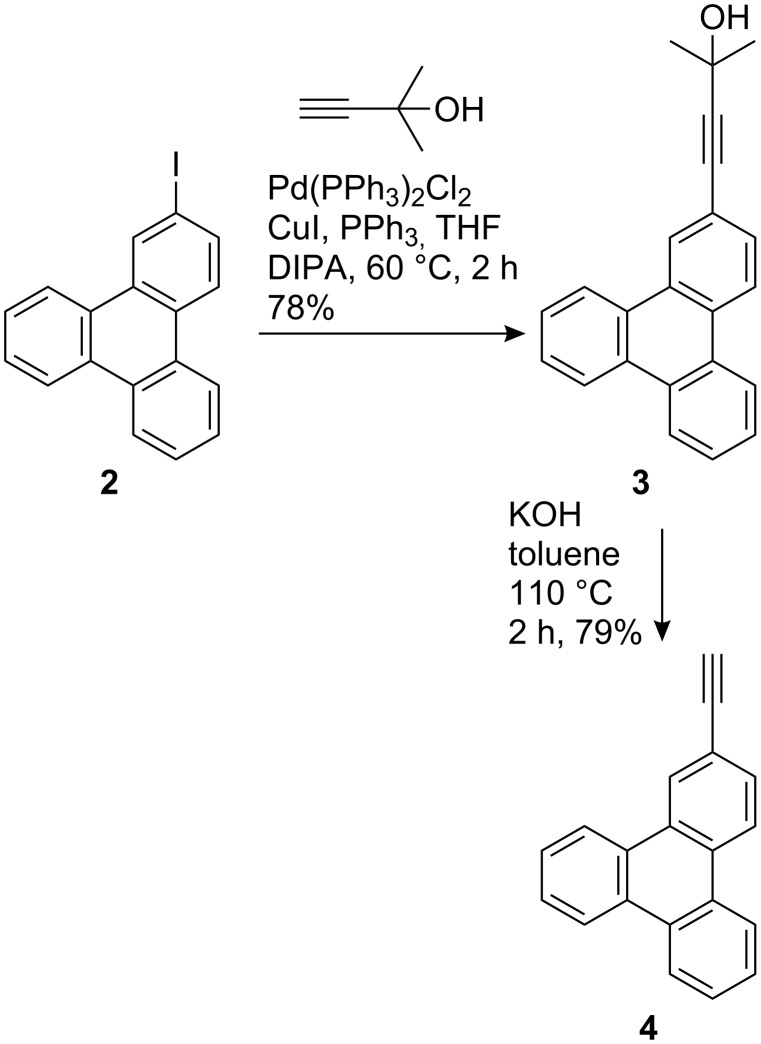
Synthesis of 2-ethynyltriphenylene (**4**).

**Scheme 3:**
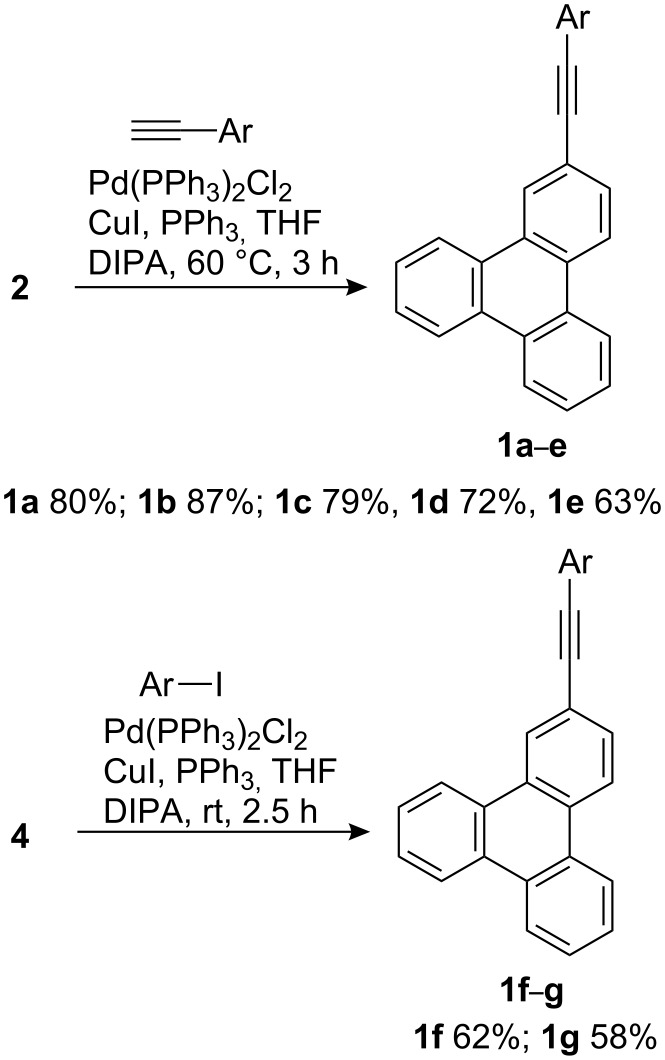
Synthesis of phenylethynyltriphenylene derivatives **1a**–**g**.

2-Ethynyltriphenylene (**4**) was synthesized by coupling 2-iodotriphenylene (**2**) with 1,1-dimethylpropargyl alcohol followed by deprotection with KOH in refluxing toluene (Scheme 2). 2-Phenylethynyltriphenylene (**1a**) and those bearing electron withdrawing substituent (**1b**–**e**) were synthesized by the Sonogashira coupling of 2-iodotriphenylene (**2**) with the corresponding phenylacetylene derivatives. Although **1f**–**g** were also synthesized by this procedure, their purification proved difficult due to an inseparable minor product formed in these reactions. Therefore compounds **1f**–**g** were synthesized via the Sonogashira coupling of 2-ethynyltriphenylene (**4**) and the corresponding iodoarenes (Scheme 3). Compounds **1a**–**f** were obtained as colorless solids in good yields. Derivative **1g** was obtained as an orange solid in 58% yield. All compounds were purified by column chromatography and thoroughly characterized by various spectroscopic methods and analytical data.

### Absorption and fluorescence emission studies

**Figure 1:**
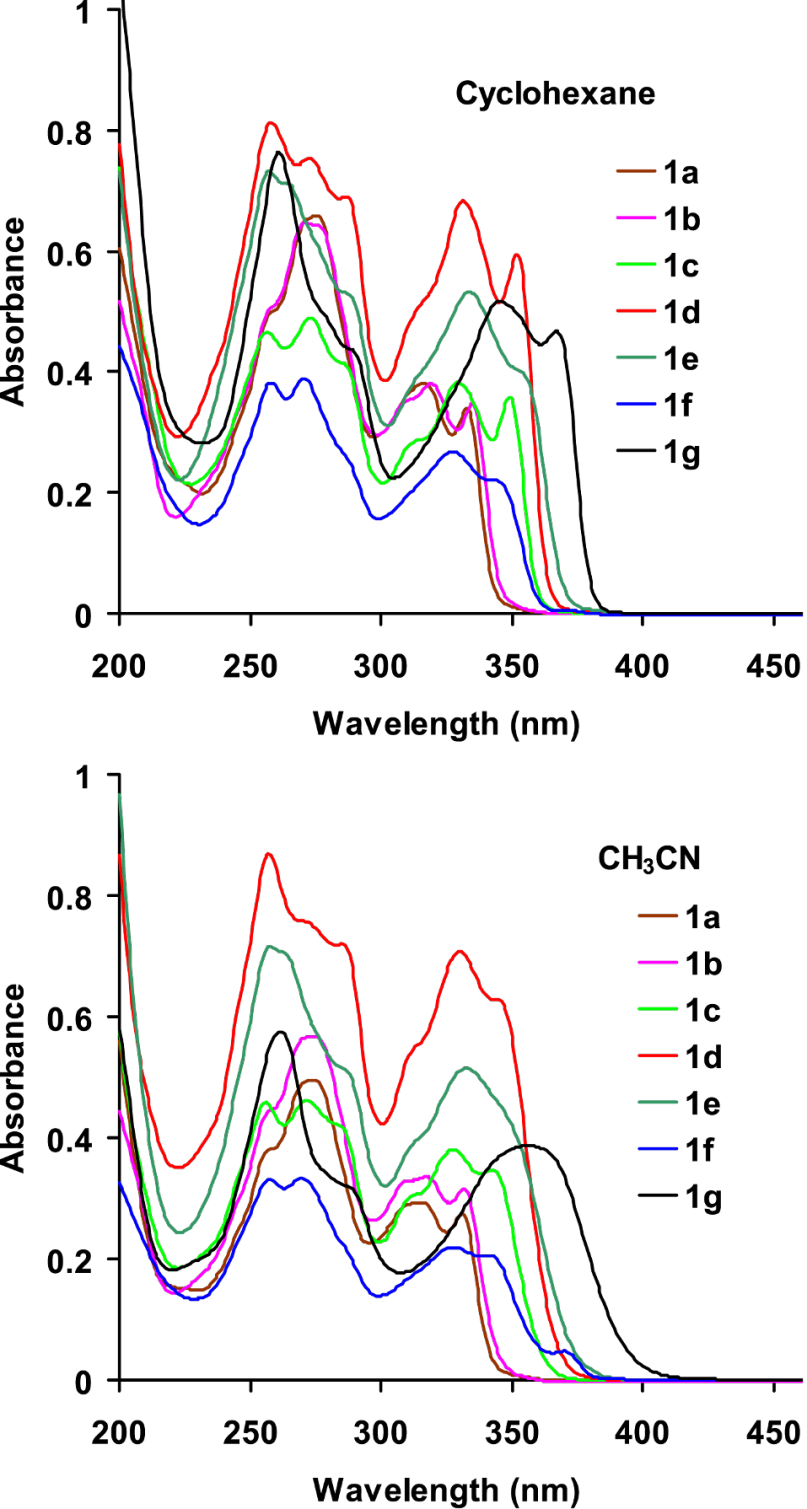
Absorption spectra of **1a**–**g** in cyclohexane and acetonitrile (10^−5^ M).

The UV–vis absorption spectra of **1a**–**g** were recorded in various solvents ranging from non-polar cyclohexane to dipolar aprotic DMSO to polar protic ethanol and isopropanol. The absorption spectra of **1a**–**g** in cyclohexane and acetonitrile are shown in Figure 1. The lowest energy absorption of triphenylene is symmetry forbidden and appears at 330–340 nm [23]. Compared to triphenylene, the absorption bands of derivatives **1a**–**g** are consistently red shifted and more intense (symmetry allowed) due to extended conjugation with the phenylethynyl group and loss of symmetry, respectively. Compared to **1a** and **1b**, the lowest energy absorption bands of **1c**–**g** are further red shifted in both cyclohexane and acetonitrile. Although there is no significant solvent effect on the absorption bands of **1a**–**g** in these two solvents, the lowest energy absorption bands are red shifted by 5–10 nm in cyclohexane compared to acetonitrile. Moreover, the vibrational fine structure is more clearly seen in cyclohexane than in acetonitrile where the bands are relatively broadened, especially for **1g**. These observations led to the conclusion that irrespective of the substituents on the phenyl ring, the ground state of these molecules is relatively non-polar and devoid of significant solvent and substituent effects. By contrast, the fluorescence emission bands of **1a**–**g** showed significant substituent and solvent effects.

**Figure 2:**
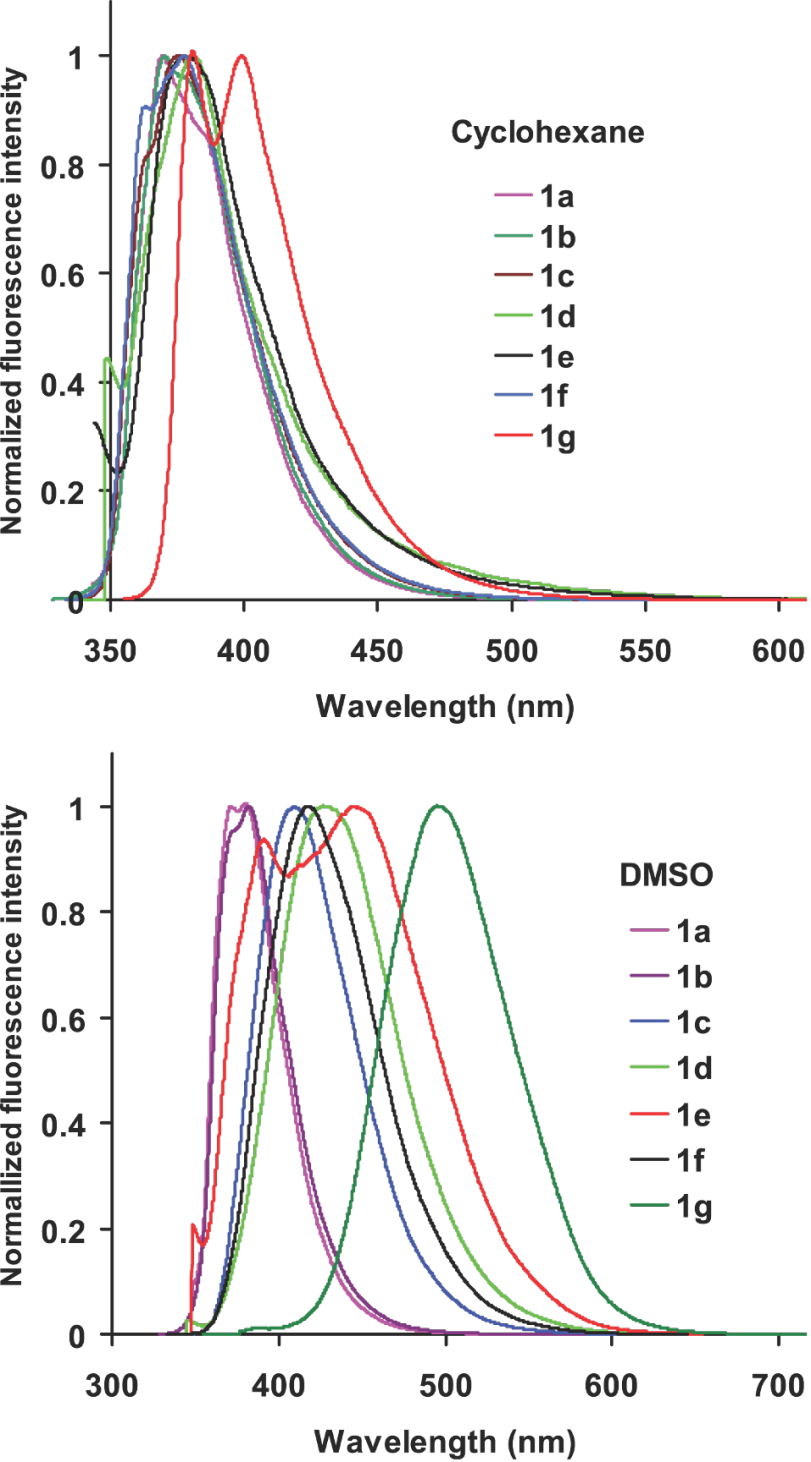
Fluorescence emission spectra of **1a**–**g** in cyclohexane and DMSO (10^−5^ M), λ_ex_ = 335 nm.

**Figure 3:**
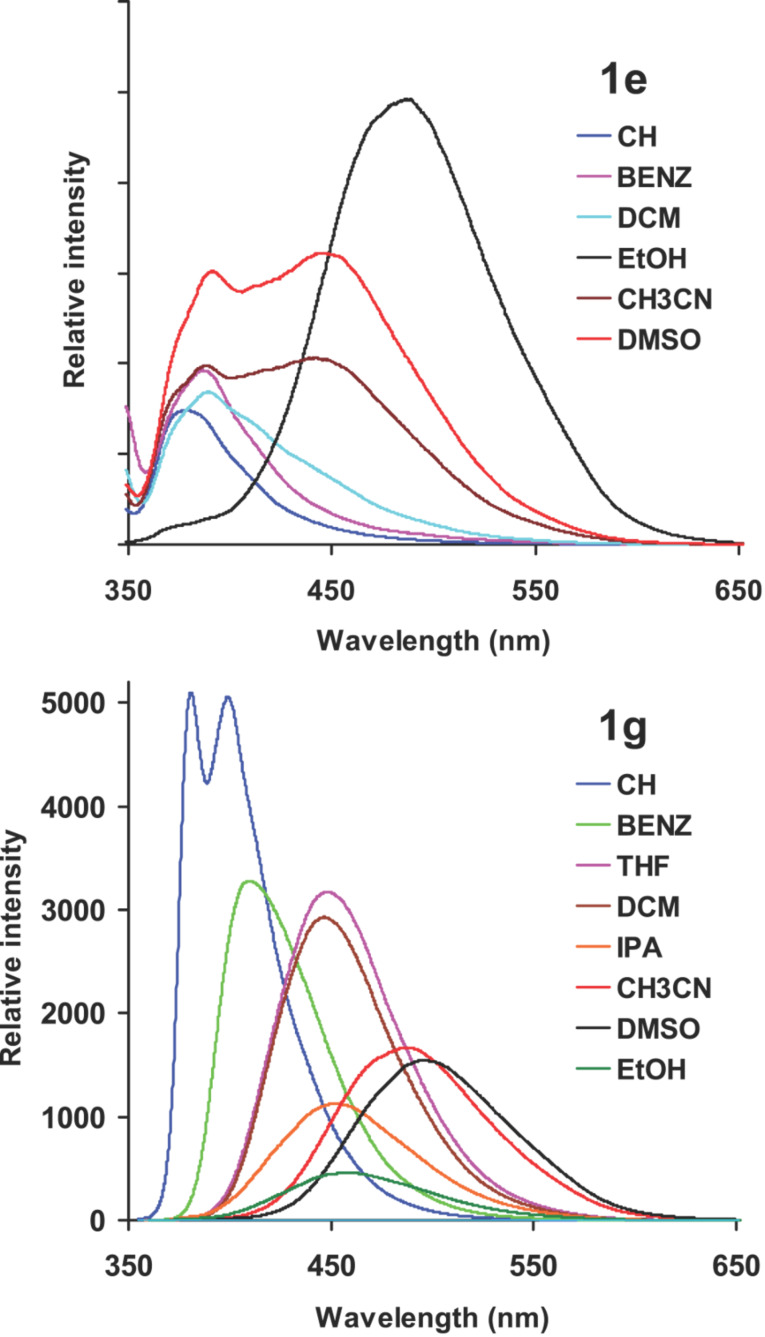
Fluorescence emission spectra of **1e** and **1g** in various solvents (10^−5^ M). CH – cyclohexane (λ_ex_ = 334 nm (**1e**) and 345 nm (**1g**)), BENZ – benzene (λ_ex_ = 338 nm (**1e**) and 335 nm (**1g**)), DCM – dichloromethane (λ_ex_ = 336 nm (**1e**) and 357 nm (**1g**)), IPA – isopropyl alcohol CH_3_CN (λ_ex_ = 348 nm (**1g**)), DMSO (λ_ex_ = 338 nm (**1e**) and 367 nm (**1g**). EtOH (λ_ex_ = 335 nm (**1e** and **1g**)), THF (λ_ex_ = 356 nm (**1g**)).

The fluorescence emission spectra of **1a**–**g** in cyclohexane, and DMSO are shown in Figure 2. In cyclohexane, **1a**–**f** showed emission maxima around 380 nm which is characteristic of the local excited state emission of the phenylethynyltriphenylene chromophore. The emission maximum was independent of the substituent, which implied that there was no significant ICT in the excited state in cyclohexane. In the case of **1g**, two emission bands were observed at 381 and 400 nm. The 381 nm band was assigned to emission from the local excited state of the phenylethynyltriphenylene chromophore and the band at 400 nm to ICT from the dimethylamino group to triphenylene moiety [36]. In DMSO, the fluorescence emission of **1a**–**b** appeared at around 380 nm, arising from the local excited state of phenylethynyltriphenylene chromophore. The emission maxima of all the other substrates (**1c**–**g**) were progressively red shifted and highly dependent on the substituent present. The maximum red shift (499 nm) was observed in case of **1g** where excited state ICT is expected to be efficient due to the strong electron donating nature of the –NMe_2_ group. Compounds **1c**–**e** bearing electron withdrawing substituents also showed progressive red shifts (**1c**, 412 nm; **1d**, 430 nm and **1e**, 448 nm) of the emission band due to ICT where charge transfer from triphenylene moiety to the phenyl group bearing the electron withdrawing substituent occurred. In case of **1e**, two emission bands were observed at 394 and 448 nm due emission from local excitation of the triphenylene chromophore and the ICT transition, respectively. The fluorescence emission spectra of **1e** and **1g** measured in various solvents are shown in Figure 3. These two substrates, one with an electron withdrawing substituent (**1e**) and the other with an electron donating substituent (**1g**) are highlighted here because of the maximum Stokes shifts of the ICT band observed with these two compounds. With increasing solvent polarity the fluorescence maxima remained unchanged in the cases of **1a** and **1b**, whereas in all the other cases a progressive red shift of emission maxima was observed. The emission maxima of compounds **1a** (with no substituent in the phenyl ring) and **1b** (with a CF_3_ substituent ) were unaffected by a change of solvent polarity, whereas in derivatives **1c**–**g** the substituent had a strong effect on the emission maxima with increasing solvent polarity.

**Figure 4:**
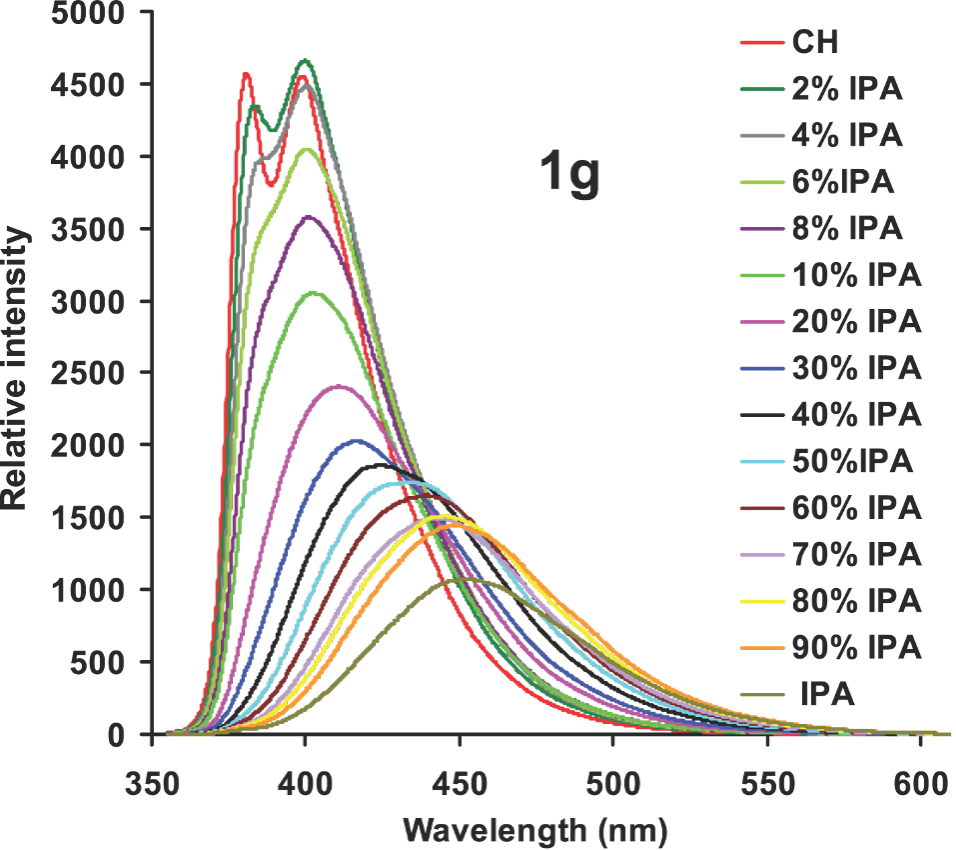
Effect of binary solvent system (cyclohexane-isopropyl alcohol) on the fluorescence emission of **1g** (10^−5^ M). CH – cyclohexane, IPA – isopropyl alcohol, λ_ex_ = 335 nm.

In cases of **1c** and **1f**–**g**, the maximum Stokes shift was observed in DMSO where as in case of **1d**–**e** the maximum Stokes shift was observed in ethanol. Compounds **1d**–**e** are carbonyl derivatives and the maximum shift in hydroxylic solvents might be due to strong hydrogen bonding interactions between the solvent and the carbonyl group in the excited state. In order to probe further the effect of solvent polarity on the emission maximum (solvatochromic fluorescence emission), the fluorescence spectrum of **1g** was recorded in binary solvent mixtures of cyclohexane and isopropyl alcohol. Initially in cyclohexane, two emission bands were observed. With the addition of isopropyl alcohol the emission maximum shifted to longer wavelengths and the two bands merged. In an approximately 10% isopropyl alcohol-cyclohexane mixture, the band that was assigned to emission from a local excited state vanished and only a band due to ICT was observed (Figure 4). With increasing solvent polarity the emission intensity decreased due to competing excited electron transfer quenching of fluorescence.

### Correlation of Stokes shifts with solvent polarity

**Figure 5:**
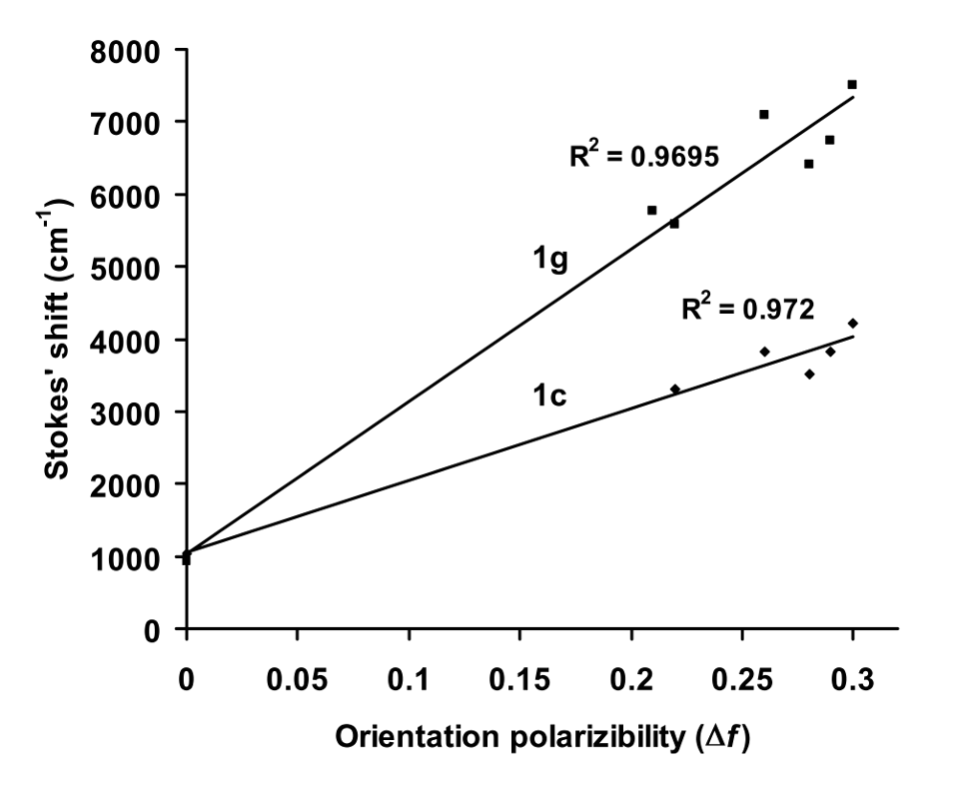
Lippert–Mataga plot showing Stokes shift as a function of solvent orientation polarizibility (Δ*f*).

**Figure 6:**
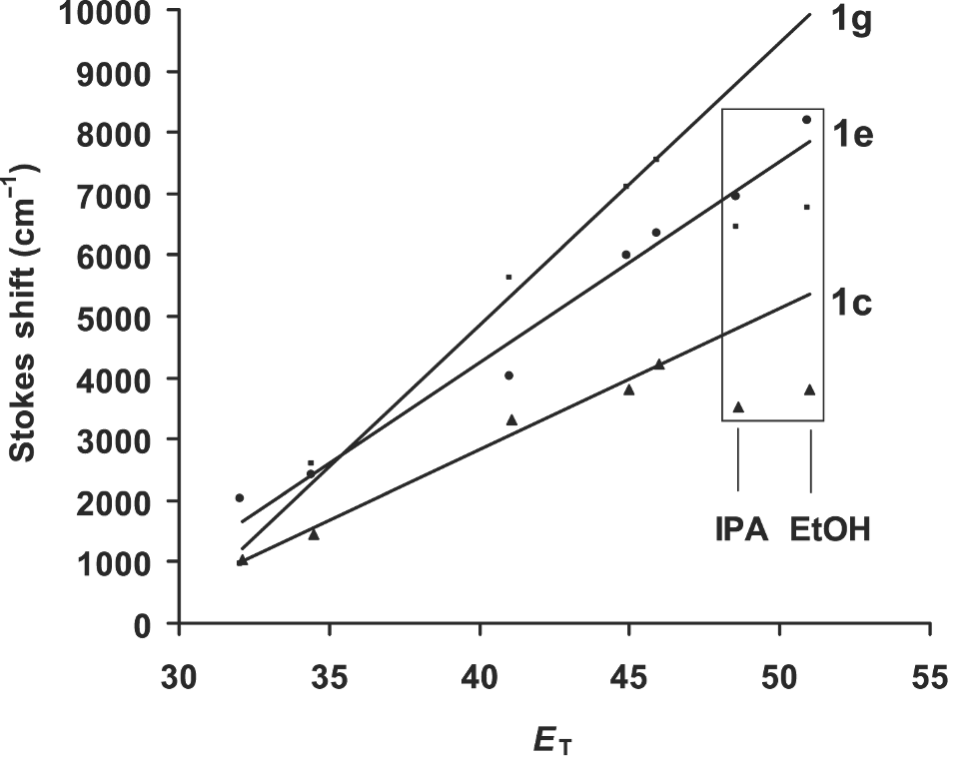
Correlation of Stokes shift with *E*_T_(30) scale.

Solvent induced spectral shifts are often interpreted in terms of the Lippert–Mataga [43–45] equation, which describes Stokes shifts in terms of the change in the dipole moment of the fluorophore and the dependence of the energy of the dipole on the dielectric constant and refractive index of the solvent. The Lippert–Mataga equation accounts for the general solvent effect and does not account for specific solvent–fluorophore interactions, for example, through hydrogen bonding etc. The Lippert–Mataga plot for **1c** and **1g** are shown in Figure 5 as representative examples. From the slope of these plots the change in the dipole moment (Δμ) of the fluorophore upon electronic excitation (μ_ES_ − μ_GS_) was estimated assuming the molecular radius as the cavity radius [11]. The molecules under consideration are non-spherical in nature. Hence, the above assumption of substituting molecular radius for cavity radius is only approximate. The molecular radii for **1c** (8.8 Å)**, 1e** (10.2 Å) and **1g** (9.1 Å) were obtained from semi-empirical AM1 calculations [46]. The change in the dipole moments (Δμ) were, 25.7 D for **1c**, 43.0 D for **1e** and 40.0 D for **1g**, respectively (Table 1). The change in dipole moment for **1g** is higher than that of the corresponding pyrene derivative (30 D) [36]. Whenever there is an excited state charge transfer process, Reichardt–Dimroth’s *E*_T_(30) [47–48] scale is more useful to correlate the solvent induced Stokes shift. Correlation using *E*_T_(30) scale often follows two distinct lines, one for the non-protic solvents and other for the protic solvents. The data points corresponding to ethanol and isopropyl alcohol are indicated in Figure 6. In protic solvents specific solvent–fluorophore interaction such as hydrogen bonding is possible and the extent of this interaction would depend upon the functional groups present in the fluorophore. In the case of **1e** all the data points of the correlation between Stokes shift and *E*_T_(30) lie on the straight line with a correlation coefficient of 0.98. A similar trend is observed in the case of **1d**. However, in cases of **1c** and **1g**, the data points corresponding to the protic solvents do not lie on the straight line since the observed Stokes shifts are much lower than expected for these solvents. Derivative **1e** is a carbonyl compound and stabilization due to hydrogen bonding interaction with protic solvents is expected both in the ground and excited state. Such a specific solvent-fluorophore interaction might be weak in the case of other derivatives. Thus, the correlation of Stokes shift with *E*_T_(30) helps in identifying specific solvent–fluorophore interactions.

### Quantum yield of fluorescence

From Figure 3 and Figure 4 it is clear that with increasing solvent polarity, the intensity of emission decreases along with the bathochromic shift of the wavelength of emission. The fluorescence intensity of molecular systems that undergo efficient ICT upon photoexcitation decreases due to competing electron transfer from the donor to the acceptor site that quenches the fluorescence [49–51]. For compounds **1c**, **1f** and **1g**, the fluorescence quantum yield decreased upon changing the solvent from cyclohexane to DMSO (Table 1). For compounds **1a** and **1b**, which did not show strong solvatochromic emission, the reverse trend was observed. The quantum yield of fluorescence increased in DMSO compared to cyclohexane. This might be due to the increase in the viscosity of the medium which quenches the non-radiative pathways. In the cases of the carbonyl derivatives **1d** and **1e**, the quantum yield of fluorescence was measured in ethanol. The fluorescence quantum yields for these derivatives in ethanol were low, presumably due to facile electron transfer and hydrogen bonding interaction with the solvent which enhances the non-radiative processes. In polar solvents electron transfer from the aromatic moiety to the benzophenone has been previously shown by time resolved spectroscopy to result in the formation of a radical ion pair [52–54].

**Table 1:** Representative absorption, emission and fluorescence quantum yield data.

Substrate	Solvent^a^	Absorption λ_max_ (nm)	Emission λ_max_ (nm)	Φ_f_^b^	Δμ (D)^c^

**1a**	CH DMSO	334 336	370 371	0.20 0.28	
**1b**	CH DMSO	334 337	368 373	0.17 0.35	
**1c**	CH DMSO	349 353	362 412	0.60 0.54	25.7
**1d**	CH EtOH	351 353	383 434	— 0.32	29.5
**1e**	CH EtOH	354 350	381 490	— 0.11	43
**1f**	CH DMSO	343 348	365 421	0.58 0.43	28
**1g**	CH DMSO	367 367	380 499	0.99 0.36	40

^a^CH = cyclohexane, ^b^fluorescence quantum yield relative to quinine sulfate standard, ^c^change in dipole moment (μ_ES_ − μ_GS_) due to excited state ICT calculated from Lippert–Mataga plot.

### HOMO and LUMO surfaces and energy gaps

**Figure 7:**
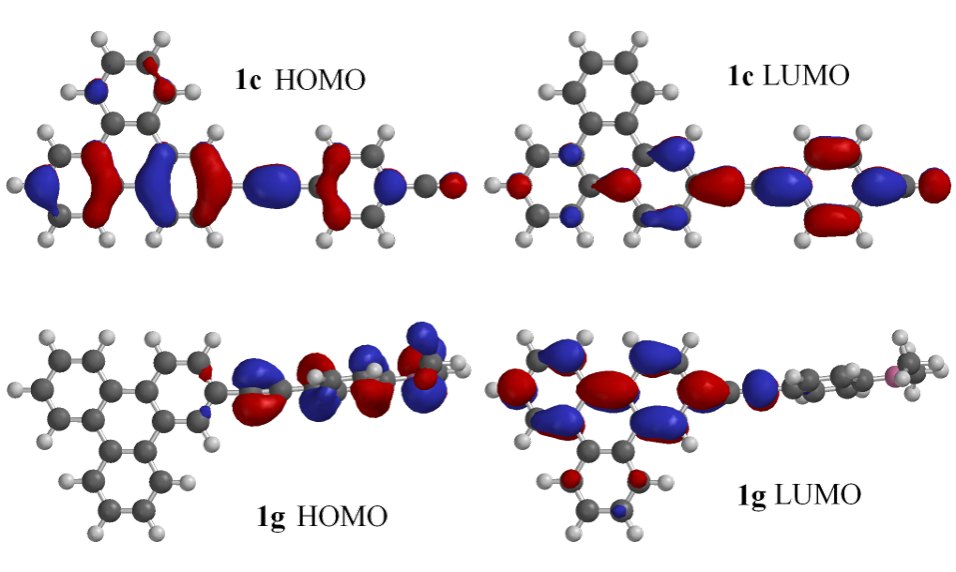
HOMO and LUMO surfaces of **1c** and **1g** according to DFT calculations.

Cyclic voltammograms of **1a**–**e** and **1g** were recorded in acetonitrile in order to obtain the redox potentials, as well as to estimate the HOMO–LUMO gap of these derivatives [31–35]. All the compounds showed a single irreversible oxidation peak and multiple reduction peaks. The HOMO–LUMO gap was estimated from the CV data as the difference between the oxidation peak potential and the reduction peak potential (Table 2) and compared with the HOMO–LUMO gap estimated from the onset of optical absorption from the UV–vis spectra in acetonitrile. The HOMO–LUMO gaps obtained by these two methods are comparable considering the approximate nature of these methods of estimation. The optimized structures, HOMO and LUMO surfaces of **1c** and **1g** were obtained by DFT calculations [46]. In case of **1c**, the molecule is planar whereas **1g** is twisted. The dihedral angle between the plane of the triphenylene ring and the plane of the phenyl ring is 96° for **1g**. These observations are comparable with the geometry of the corresponding pyrene derivatives reported earlier [31–32]. The HOMO and LUMO surfaces are shown in Figure 7. In case of **1g**, the HOMO density is mainly located on the dimethylaminophenylethynyl moiety and the triphenylene moiety is devoid of any HOMO density. The LUMO of **1g** is mainly located on the triphenylene moiety indicating that the dimethylaminophenylethynyl group is the donor and the triphenylene group the acceptor. In case of **1c** the situation is reversed, the HOMO density is located mainly on the triphenylene moiety and the LUMO density is on the cyanophenylethynyl group. This indicates that there is role reversal of the triphenylene moiety either as a donor or as an acceptor depending upon the nature of the functional group attached to the phenylethynyl unit. These findings are consistent with the earlier reports on the pyrene derivatives [31].

**Table 2:** HOMO-LUMO energy gap and change in dipole moment due to ICT.

Substrate	Δ*E* (eV)^a^	*E*_p(ox)_ (V)^b^	*E*_p(red)_ (V)^b^	Δ*E* (eV)^c^

**1a**	3.42	+1.60	−2.14	3.74
**1b**	3.41	+1.65	−2.02	3.67
**1c**	3.30	+1.68	−1.85	3.53
**1d**	3.27	+1.66	−1.70	3.36
**1e**	3.20	+1.63	−1.63	3.26
**1f**	3.19	—	—	—
**1g**	2.97	+0.76	−2.27	3.03

^a^HOMO–LUMO energy gap estimated on the basis of absorption data, ^b^at 0.1 Vs^−1^ scan rate, ^c^HOMO–LUMO energy gap estimated on the basis of electrochemical data.

## Conclusion

Several 2-phenylethynyltriphenylene derivatives bearing electron donating and electron releasing groups on the phenyl ring were synthesized. Their absorption and fluorescence emission were studied in several solvents. The absorption maximum of these derivatives was not siginificantly altered by solvent polarity. However, the fluorescence emission maxima showed strong solvent polarity dependence and large Stokes shifts were observed. These observations are explained on the basis of an excited state intramolecular charge transfer (ICT) process. Derivative **1g**, with dimethylamino substituent, showed the maximum solvent effect with a Stokes shift of nearly 130 nm (7828 cm^−1^) in DMSO in comparison to that observed in cyclohexane. Derivatives bearing carbonyl substituents (**1d**–**e**) showed large Stokes shift in polar protic solvents such as ethanol and isopropyl alcohol, presumeably due to the hydrogen bonding stabilization of the excited state by these solvents. The Stokes shifts were correlated with solvent orientation polarizibility by the Lippert–Mataga equation and Reichardt’s *E*_T_(30) solvent polarity scale. HOMO–LUMO gaps were calculated from both optical and electrochemical data. HOMO and LUMO surfaces based on DFT calculations show that the triphenylene chromophore can act either as an electron donor or as an electron acceptor in the ICT process, depending upon the nature of substituent on the phenyl ring. Derivatives **1e** and **1g** are potential candidates for use as solvent polarity probes. However, their performance is only comparable to those of the corresponding pyrene derivatives.

## Experimental

**Synthesis of 2-methyl-4-(triphenylen-2-yl)but-3-yn-2-ol (3).** A Schlenk flask was charged with a mixture of 2-iodotriphenylene and triphenylene (2.0 g, 45:55 by ^1^H NMR) (see Supporting Information File 1), Pd(PPh_3_)_2_Cl_2_ (0.2 g, 0.3 mmol), PPh_3_ (0.15 g, 0.6 mmol), CuI (0.105 g, 0.6 mmol), degassed THF (30 mL) and diisopropylamine (30 mL). The mixture was stirred at room temperature for 15 min and 2-methyl-3-butyn-2-ol (0.32 g, 3.8 mmol) was added. Stirring was continued for 2 h at 60 °C after which time the solvent was removed and the residue dissolved in dichloromethane (100 mL). The solution was washed successively with 5% aq HCl (2 × 60 mL) and water (60 mL). The organic layer was dried over anhydrous sodium sulfate and solvent removed under reduced pressure. The crude product was purified by column chromatography on silica gel. Elution with hexane to remove unreacted triphenylene followed by elution with a mixture of hexane and ethyl acetate (9:1, v/v) gave **3** as a colorless solid (0.95 g, 78%), mp 153–155 °C; IR (neat) 3331, 2978, 2212 cm^−1^; ^1^H NMR (CDCl_3_, 400 MHz) δ_H_ = 8.71 (d, *J* = 1.2 Hz, 1H), 8.53–8.63 (m, 5H), 7.63–7.67 (m, 5H), 2.24 (s, 1H), 1.72 (s, 6H) ppm. ^13^C NMR (CDCl_3_, 100 MHz) δ_c_ = 130.0, 129.9, 129.6, 129.5, 129.2, 129.0, 127.6, 127.5, 1273, 126.9, 123.5, 123.4, 123.38, 123.31, 123.27, 121.3, 94.5, 82.5, 65.8, 31.6 ppm. ESI Q-TOF MS *m/z* 333 [M + Na]^+^, 293 [M − OH]^+^; HRMS calcd for C_23_H_18_ONa [M − Na]^+^ 333.1255; found, 333.1257.

**Synthesis of 2-ethynyltriphenylene (4).** To a degassed solution of **3** (0.79 g, 2.5 mmol) in toluene (100 mL), KOH (0.57 g, 10.2 mmol) was added and the reaction mixtureheated under reflux for 2.5 h. Upon completion of the reaction, the hot reaction mixture was filtered and the residue washed with toluene (10 mL). The combined filtrate and washings were washed with water (2 × 60 mL). The organic layer was separated and dried over anhydrous sodium sulfate. The solvent was removed under reduced pressure and the crude product purified by column chromatography on silica gel with hexane and dichloromethane (95:5, v/v) as eluant to give **4** as a colorless solid (0.508 g, 79%); mp 149–151 °C; IR (neat) 3280, 2194 cm^−1^; ^1^H NMR (CDCl_3_, 400 MHz) δ_H_ = 8.80 (d, *J* = 1.2 Hz, 1H), 8.57–8.65 (m, 5H), 7.74 (dd, *J* = 1.2, 8.8 Hz, 1H), 7.65–7.68 (m, 4H), 3.23 (s, 1H) ppm; ^13^C NMR (CDCl_3_, 100 MHz) δ_c_ 130.2, 130.1, 129.9, 129.1, 128.9, 127.7, 127.6, 127.5, 127.4, 127.3, 123.5, 123.4, 123.3, 120.6, 84.1, 77.9 ppm; MALDI-TOF MS *m/z* (%) 252 (72) [M^+^], 253 (100) [M^+^ + 1], 254 (22) [M^+^ + 2]; Anal. calcd. for C_20_H_12_ C, 95.23; H, 4.75. Found C, 95.04, H 4.60.

**General procedure for the synthesis of 1a**–**e. 1a**–**e** were synthesized by coupling 2-iodotriphenylene (2 mmol) with the corresponding arylethyne (1.9 mmol) (see Scheme 3) according to the procedure described above for the synthesis of **3**.

**2-(Phenylethynyl)triphenylene (1a).** The crude product was purified by column chromatography with hexane as eluant to afford **1a** as a colorless solid (0.495 g, 80%), mp 180–182 °C; IR (neat) 3069, 2217 cm^−1^; ^1^H NMR (CDCl_3_, 500 MHz) δ_H_ = 8.80 (d, *J* = 1.5 Hz, 1H), 8.55–8.64 (m, 5H), 7.75 (dd, *J* = 1.5, 8.5 Hz, 1H), 7.61–7.66 (m, 6H), 7.36-7.40 (m, 3H) ppm; ^13^C NMR (CDCl_3_, 125 MHz) δ_c_ 131.7, 130.1, 130.0, 129.9, 129.7, 129.6, 129.3, 129.1, 128.5, 128.4, 127.59, 127.58, 127.4, 127.3, 126.8, 123.5, 123.43, 123.41, 123.36, 123.31, 121.9, 90.2, 89.9 ppm; The mass spectrum was recorded as the silver ion adduct of **1a** by adding silver triflate to a solution of **1a** in acetonitrile prior to measurement. ESI Q-TOF MS *m/z* 435 [M + Ag]^+^ along with the isotope peaks in the expected intensity ratios; HRMS calcd for C_26_H_16_Ag [M + Ag]^+^ 435.0303; found, 435.0298.

**2-(3-Trifluoromethylphenylethynyl)triphenylene (1b).** The crude product was purified three times by column chromatography with hexane as eluant to yield **1b** as a colorless solid (0.654 g, 87%), mp 137–139 °C; IR (neat) 3069, 2213 cm^−1^; ^1^H NMR (CDCl_3_, 500 MHz) δ_H_ = 8.80 (d, *J* = 1.5 Hz, 1H), 8.55–8.68 (m, 5H), 7.8 (m, 1H), 7.73–7.77 (m, 2H), 7.59–7.67 (m, 5H), 7.48-7.51 (m, 1H) ppm; ^13^C NMR (CDCl_3_, 125 MHz) δ_c_ 134.7, 131 (q, ^2^*J*_C-F_ = 32.5 Hz), 130.1, 130.0, 129.9, 129.8, 129.7, 129.2, 129.0, 128.9, 128.5 (q, ^3^*J*_C-F_ = 3.75 Hz), 127.7, 127.6, 127.41, 127.39, 127.0, 124.87 (q, ^3^*J*_C-F_ = 3.75 Hz), 124.3, 123.5, 123.4, 123.3, 122.7, 121.1, 91.4, 88.6 ppm; MALDI-TOF MS C_27_H_15_F_3_ *m/z* (%) 396 (100) [M^+^], 397 (84) [M^+^ + 1], 398 (22) [M^+^ + 2].

**4-(2-Triphenylenylethynyl)benzonitrile (1c).** The crude product was purified by column chromatography with a mixture of hexane and dichloromethane (85:15, v/v) as eluant to yield **1c** as a colorless solid (0.529 g, 79%), mp 192–194 °C; IR (neat) 3059, 2221 cm^−1^; ^1^H NMR (CDCl_3_, 400 MHz) δ_H_ = 8.82 (d, *J* = 1.2 Hz, 1H), 8.60–8.66 (m, 5H), 7.77 (dd, *J* = 1.2, 8.8 Hz, 1H), 7.66–7.69 (m, 8H) ppm;^13^C NMR (CDCl_3_, 100 MHz) δ_c_ 132.1, 130.2, 130.0, 129.8, 129.1, 128.9, 128.2, 127.9, 127.8, 127.4, 127.2, 123.6, 123.5, 123.4, 123.37, 123.3, 120.8, 119.6, 111.5, 94.3, 88.8 ppm; ESI Q-TOF MS C_27_H_15_N *m/z* 376 [M +Na]^+^, 354 [M + H]^+^; HRMS calcd for C_27_H_16_N [M + H]^+^ 354.1283; found, 354.1287.

**4-(2-Triphenylenylethynyl)acetophenone (1d).** The crude product was purified by column chromatography with a mixture of hexane and dichloromethane (4:1, v/v) as eluant to yield **1d** as a colorless solid (0.506 g, 72%), mp 181–183 °C; IR (neat) 3067, 2218, 1669 cm^−1^; ^1^H NMR (CDCl_3_, 400 MHz) δ_H_ = 8.86 (d, *J* = 1.6 Hz, 1H), 8.62–8.68 (m, 5H), 7.97–8.0 (m, 2H), 7.80 (dd, *J* = 1.6, 8.8 Hz, 1H), 7.66–7.71 (m, 6H), 2.64 (s, 3H) ppm; ^13^C NMR (CDCl_3_, 100 MHz) δ_c_ 191.3, 136.6, 131.8, 130.2, 130.0, 129.9, 129.8, 129.2, 129.0, 128.4, 128.2, 127.8, 127.7, 127.4, 127.1, 123.6, 123.4, 121.3, 93.2, 89.4, 26.6 ppm; ESI Q-TOF MS *m/z* 371 [M + H]^+^; HRMS calcd for C_28_H_19_O [M + H]^+^ 371.1436; found, 371.1433.

**4-(2-Triphenylenylethynyl)benzophenone (1e).** The crude product was purified by column chromatography with a mixture of hexane and dichloromethane (80:20, v/v) as eluant to yield **1e** as a colorless solid (0.517 g, 63%), mp 172–174 °C; IR (neat) 3080, 2211, 1658 cm^−1^; ^1^H NMR (CDCl_3_, 400 MHz) δ_H_ = 8.85 (d, *J* = 1.2 Hz, 1H), 8.61–8.66 (m, 5H), 7.79–7.86 (m, 5H), 7.59–7.73 (m, 7H), 7.49–7.53 (m, 2H) ppm; ^13^C NMR (CDCl_3_, 100 MHz) δ_c_ 196.0, 136.9, 132.6, 131.5, 130.20, 130.16, 130.06, 130.03, 129.9, 129.8, 128.4, 127.8, 127.7, 127.5, 127.1, 123.6, 123.5, 123.4, 121.4, 93.0, 89.5 ppm; ESI Q-TOF MS *m/z* 433 [M + H]^+^; HRMS calcd for C_33_H_21_O [M + H]^+^ 433.1592; found, 433.1593.

**General procedure for the synthesis of 1f**–**g.** A Schlenk flask was charged with the corresponding aryl iodide (0.6 mmol), Pd(PPh_3_)_2_Cl_2_ (0.011 g, 0.015 mmol), PPh_3_ (0.008 g, 0.03 mmol), CuI (0.006 g, 0.03 mmol), degassed THF (30 mL) and diisopropylamine (10 mL). The reaction mixture was stirred at room temperature for 15 min and a solution of 2-ethynyltriphenylene (**4**) (0.5 mmol) in THF (3mL) added dropwise. Stirring was continued for 2.5 h. Removal of solvent and other volatile materials under reduced pressure gave the crude product which was purified by column chromatography on silica gel.

**2-(3,4-Bis(decyloxy)phenylethynyl)triphenylene (1f).** The crude product was purified by column chromatography with a mixture of hexane and dichloromethane (85:15, v/v) as eluant to yield **1f** as a colorless solid (0.201 g, 62%), mp 118–120 °C; IR (neat) 3084, 2959, 2918, 2850 cm^−1^; ^1^H NMR (CDCl_3_, 400 MHz) δ_H_ = 8.82 (d, *J* = 1.2 Hz, 1H), 8.58–8.65 (m, 5H), 7.78 (dd, *J* = 1.6, 8.4 Hz, 1H), 7.64–7.68 (m, 4H), 7.20 (dd, *J* = 1.2 Hz, 8.8 Hz, 1H), 7.15 (d, *J* = 1.6 Hz, 1H), 6.87 (d, *J* = 8.4 Hz, 1H), 4.05 (qt , J = 7.2, 8.4 Hz, 4H), 1.83–1.88 (m, 4H), 1.47–1.52 (m, 4H), 1.27–1.29 (m, 24H), 0.88-0.92 (m, 6H) ppm; ^13^C NMR (CDCl_3_, 100 MHz) δ_c_ 149.9, 148.9, 130.0, 129.9, 129.8, 129.4, 129.3, 229.2, 127.5, 127.3, 126.6, 125.1, 123.5, 123.4, 123.35, 123.3, 122.3, 116, 115.4, 113.5, 113.5, 90.6, 88.2, 69.4, 69.2, 31.9, 29.7, 29.6, 29.4, 29.36, 29.30, 29.26, 26.1, 22.7, 14.1 ppm; MALDI-TOF MS: *m/z* (%) 640 (100) [M^+^], 641 (49) [M^+^ + 1], 642 (9) [M^+^ + 2].

***N,N*-Dimethyl-4-(2-triphenylenylethynyl)aniline (1g).** The crude product was purified by column chromatography with a mixture of hexane and dichloromethane (9:1, v/v) as eluant to yield **1g** as an orange solid (0.108 g, 58%), mp 212–214 °C (decomposed during melting); IR (neat) 3064, 2798, 2190 cm^−1^; ^1^H NMR (CDCl_3_, 400 MHz) δ_H_ = 8.80 (d, *J* = 1.2 Hz, 1H), 8.61–8.66 (m, 4H), 8.59 (d, *J* = 8.4 Hz, 1H), 7.77 (dd, *J* = 1.6, 8.4 Hz, 1H), 7.65–7.69 (m, 4H), 7.51 (d, *J* = 9.2 Hz, 2H), 6.71 (d, *J* = 9.2 Hz, 2H), 3.02 (s, 3H) ppm; ^13^C NMR (CDCl_3_, 100 MHz) δ_c_ 150.2, 132.8, 130.0, 129.9, 129.8, 129.7, 129.3, 128.9, 127.5, 127.4, 127.3, 126.3, 123.5, 123.4, 123.3, 123.2, 122.9, 111.9, 110.0, 91.6, 87.9, 40.2 ppm; ESI Q-TOF MS *m/z* 372 [M + H]^+^; HRMS calcd for C_28_H_22_N [M + H]^+^ 372.1752; found, 372.1759.

## Supporting Information

Supporting Information includes the NMR spectra of all compounds (**1a**–**g, 2**–**4**), UV–vis data in various solvents, electrochemical data for **1a**–**g** and calculated atomic coordinates for **1c** and **1g**.

File 1 Experimental data for compounds **1a**–**g** and **2**–**4**.
