# Supplementary material for: Donor-acceptor substituted phenylethynyltriphenylenes – excited state intramolecular charge transfer, solvatochromic absorption and fluorescence emission
Source: Beilstein J Org Chem. 2010 Oct 18;6:992–1001. doi: 10.3762/bjoc.6.112 (PMC2981938; doi:10.3762/bjoc.6.112)
Supplement: File 1 — Experimental data for compounds 1a–g and 2–4. [file Beilstein_J_Org_Chem-06-992-s001.pdf]

Supporting Information

for

Donor-acceptor substituted phenylethynyltriphenylenes – excited state intramolecular charge transfer, solvatochromic absorption and fluorescence emission

Ritesh Nandy and Sethuraman Sankararaman*

Address: Department of Chemistry, Indian Institute of Technology Madras, Chennai 600036, India

Email: Sethuraman Sankararaman – sanka@iitm.ac.in
* Corresponding author

Experimental data for compounds **1a–g** and **2–4**

Contents Page No

Synthesis of 2-iodotriphenylene. S4

Table 1: Absorption spectral data for **1a-g** in various solvents. S5

CV measurements. S6

Figure 1: ^{1}H NMR spectrum of **1a** in $CDCl_3$. S7

Figure 2: Expanded aromatic region of ^{1}H NMR spectrum of **1a** in $CDCl_3$. S8

Figure 3: ^{13}C NMR spectrum of **1a** in $CDCl_3$. S9

Figure 4: Expanded aromatic region of ^{13}C NMR spectrum of **1a** in $CDCl_3$. S10

Figure 5: ^{1}H NMR spectrum of **1b** in $CDCl_3$. S11

Figure 6: Expanded aromatic region of ^{1}H NMR spectrum of **1b** in $CDCl_3$. S12

Figure 7: ^{13}C NMR spectrum of **1b** in $CDCl_3$. S13

Figure 8. Expanded aromatic region of ^{13}C NMR spectrum of **1b** in $CDCl_3$. S14

Figure 9. ^{1}H NMR spectrum of **1c** in $CDCl_3$. S15

Figure 10. Expanded aromatic region of ^{1}H NMR spectrum of **1c** in $CDCl_3$. S16

Figure 11. ^{13}C NMR spectrum of **1c** in $CDCl_3$. S17

Figure 12. ^{1}H NMR spectrum of **1d** in $CDCl_3$. S18

Figure 13. Expanded aromatic region of ^{1}H NMR spectrum of **1d** in $CDCl_3$. S19

Figure 14. ^{13}C NMR spectrum of **1d** in $CDCl_3$. S20

Figure 15. ^{1}H NMR spectrum of **1e** in $CDCl_3$. S21

Figure 16. Expanded aromatic region of ^{1}H NMR spectrum of **1e** in $CDCl_3$. S22

Figure 17. ^{13}C NMR spectrum of **1e** in $CDCl_3$. S23

Figure 18. ^{1}H NMR spectrum of **1f** in $CDCl_3$. S24

Figure 19. Expanded aromatic / aliphatic regions of ^{1}H NMR spectrum of **1f** in $CDCl_3$. S25

Figure 20. ^{13}C NMR spectrum of **1f** in $CDCl_3$. S26

Figure 21. ^{1}H NMR spectrum of **1g** in $CDCl_3$. S27

Figure 22. Expanded aromatic region of ^{1}H NMR spectrum of **1g** in $CDCl_3$. S28

Figure 23. ^{13}C NMR spectrum of **1g** in $CDCl_3$. S29

Figure 24. ^{1}H NMR spectrum of **2** in $CDCl_3$. S30

Figure 25.Expanded aromatic region of ^{1}H NMR spectrum of **2** in $CDCl_3$. S31

Figure 26. ^{13}C NMR spectrum of **2** in $CDCl_3$. S32

Figure 27. Expanded aromatic region of ^{13}C NMR spectrum of **2** in $CDCl_3$. S33

Figure 28. ^{1}H NMR spectrum of **3** in $CDCl_3$. S34
Figure 29. Expanded aromatic region of ^{1}H NMR spectrum of **3** in $CDCl_3$. S35
Figure 30. ^{13}C NMR spectrum of **3** in $CDCl_3$. S36
Figure 31. ^{1}H NMR spectrum of **4** in $CDCl_3$. S37
Figure 32. Expanded aromatic region of ^{1}H NMR spectrum of **4** in $CDCl_3$. S38
Figure 33. ^{13}C NMR spectrum of **4** in $CDCl_3$. S39
DFT calculation based atomic coordinates for **1c** and **1g** S40
Figure 34. Lippert-Mataga plot for **1d** S43
Figure 35. Lippert-Mataga plot for **1f** S43

2-iodotriphenylene (2): To a suspension of triphenylene (0.300 g, 1.3 mmol) in acetic acid (50 mL), water (5 mL) and sulfuric acid (0.5 mL), was added iodine (0.200 g, 0.8 mmol) and potassium iodate (0.068 g, 0.3 mmol) (This iodination procedure has been reported earlier for pyrene, Leroy-Lhez, S.; Fages, F. *Eur. J. Org. Chem.*, **2005**, 2684–2688). The reaction mixture was heated at 55 ^{o}C for 1 day, then cooled to room temperature and poured into water (100 mL). The mixture was extracted with DCM (100 mL) and the organic layer was washed successively with sodium thiosulfate solution (2 x 50 mL) and water (100 mL), dried over anhydrous sodium sulfate and the solvent was removed under reduced pressure to give a pale brown solid. The solid was purified by column chromatography on silica gel with hexane as eluant to give an inseparable mixture of 2-iodotriphenylene and triphenylene as a white solid (0.36 g) (45:55 by ^{1}H NMR). The mixture was used as such in all further reactions. A small amount of pure 2-iodotriphenylene (**2**) was separated for characterization. ^{1}H NMR ($CDCl_3$, 400MHz): δ 8.93 (d, *J* = 1.6 Hz, 1H), 8.51-8.63 (m, 4H), 8.31 (d, *J* = 8.4 Hz, 1H), 7.89 (dd, *J* = 1.6, 8.4 Hz, 1H), 7.62-7.69 (m, 4H) ppm. ^{13}C NMR ($CDCl_3$, 100MHz): δ 135.79, 132.48, 131.75, 129.99, 129.79, 129.16, 129.06, 128.37, 127.81, 127.69, 127.42, 127.39, 125.03, 123.37, 123.33, 123.30, 123.07, 93.31 ppm. MALDI-TOF MS: m/z (%) 354 (100) [M^+], 355 (58) [M^+ + 1], 356 (10) [M^+ + 2]. An attempt to convert all of the triphenylene to 2-iodotriphenylene by using an excess of iodinating agent and a higher temperature gave a complex mixture of polyiodotriphenylenes. The above procedure is optimized to give a mixture of only monoiodotriphenylene and unreacted triphenylene. The presence of triphenylene did not cause any problem in the subsequent cross coupling reactions of this mixture and triphenylene could be easily separated by chromatography after the cross coupling reactions.

Quantum yield of fluorescence (Φ_f): Fluorescence quantum yields were measured after degassing the samples thoroughly by purging the solutions with nitrogen prior to measurement. Quinine sulfate was used as the standard (λ_{ex} = 355 nm, Φ_f = 0.57 in 0.1 M H_2SO_4). Appropriate corrections were made for the optical density of the solutions and the refractive index of the medium [1].

Table 1: Absorption spectral data for **1a-g** in various solvents.

Compound **1a**.

**Solvent**	**λ_{max} (log ε) nm**
Cyclohexane	274 (4.82), 314 (4.58), 332 (4.53)
Benzene	276 (4.87), 318 (4.59), 334 (4.52)
Dichloromethane	274 (4.79), 317 (4.56), 333 (4.52)
2-Propanol	271 (4.88), 313 (4.64), 330 (4.59)
Acetonitrile	271 (4.69), 314 (4.47), 330 (4.44)
DMSO	274 (4.88), 318 (4.64), 335 (4.59)

Compound **1b**.

**Solvent**	**λ_{max} (log ε) nm**
Cyclohexane	274 (4.81), 307 (sh, 4.53), 318 (4.58), 334 (4.54)
Benzene	275 (4.83), 308 (sh, 4.50), 321 (4.54), 336 (4.48)
Dichloromethane	275 (4.86), 309 (sh, 4.60), 319 (4.63), 334 (4.59)
2-Propanol	271 (4.85), 307 (sh, 4.59), 318 (4.61), 332 (4.56)
Acetonitrile	273 (4.75), 308 (sh, 4.51), 316 (4.52), 331 (4.50)
DMSO	275 (4.84), 310 (sh, 4.57), 322 (4.60), 337 (4.56)

Compound **1c**.

**Solvent**	**λ_{max} (log ε) nm**
Cyclohexane	255 (4.67), 272 (4.69), 285 (sh, 4.62), 310 (sh, 4.44), 328 (4.58), 348 (4.55)
Benzene	276 (4.75), 289 (sh, 4.61), 311 (sh, 4.39), 331 (4.52), 350 (4.45)
Dichloromethane	256 (4.58), 273 (4.58), 286 (sh, 4.53), 311 (sh, 4.36), 330 (4.49), 347 (4.45)
2-Propanol	255 (4.61), 270 (4.60), 286 (sh, 4.49), 310 (sh, 4.38), 328 (4.49), 344 (4.45)
Ethanol	255 (4.63), 271 (4.63), 285 (sh, 4.47), 310 (sh, 4.41), 327 (4.52), 344 (4.48)
Acetonitrile	255 (4.66), 270 (4.66), 284 (sh, 4.62), 309 (sh, 4.47), 326 (4.58), 342 (4.54)
DMSO	258 (4.68), 274 (4.65), 288 (sh, 4.60), 313 (sh, 4.42), 332 (4.54), 349 (4.49)

Compound **1d**.

**Solvent**	**λ_{max} (log ε) nm**
Cyclohexane	257 (4.91), 272 (sh, 4.88), 286 (4.84), 311 (sh, 4.68), 330 (4.85), 351 (4.77)
Benzene	275 (4.98), 288 (4.92), 313 (sh, 4.74), 334 (4.87), 353 (4.79)
Dichloromethane	258 (4.97), 275 (sh, 4.91), 287 (sh, 4.88), 313 (4.73), 333 (4.87), 350 (4.82)
2-Propanol	257 (4.96), 274 (sh, 4.89), 286 (4.86), 311 (sh, 4.71), 332 (4.85), 348 (4.80)
Ethanol	257 (4.99), 274 (sh, 4.91), 286 (4.89), 310 (sh, 4.73), 331 (4.87), 346 (4.83)
Acetonitrile	256 (4.94), 271 (sh, 4.88), 286 (4.86), 309 (sh, 4.71), 328 (4.85), 345 (4.80)
DMSO	259 (4.99), 276 (sh, 4.92), 289 (4.88), 312 (sh, 4.69), 335 (4.83), 352 (4.77).

Compound **1e**.

**Solvent**	**λ_{max} (log ε) nm**
Cyclohexane	256 (4.86), 287 (sh, 4.72), 312 (sh, 4.58), 333 (4.73), 353 (sh, 4.60)
Benzene	274 (4.75), 289 (sh, 4.71), 314 (sh, 4.54), 337 (4.68), 358 (sh, 4.56)
Dichloromethane	258 (4.83), 287 (sh, 4.68), 312 (sh, 4.52), 335 (4.67), 354 (sh, 4.59)
2-Propanol	258 (4.86), 287 (sh, 4.68), 311 (sh, 4.52), 333 (4.66), 353 (sh, 4.59)
Ethanol	258 (4.88), 287 (sh, 4.71), 310 (sh, 4.55), 333 (4.69), 353 (sh, 4.62)
Acetonitrile	256 (4.85), 286 (sh, 4.71), 310 (sh, 4.58), 331 (4.71), 350 (sh, 4.62)
DMSO	262 (4.85), 290 (sh, 4.68), 313 (sh, 4.49), 337 (4.61), 358 (sh, 4.52)

Compound **1f**.

**Solvent**	**λ_{max} (log ε) nm**
Cyclohexane	257 (4.58), 269 (4.59), 288 (sh, 4.40), 325 (4.42), 344 (4.34)
Benzene	276 (4.59), 291 (4.45), 314 (sh, 4.37), 329 (4.49), 346 (4.42)
Dichloromethane	258 (4.59), 271 (4.59), 287 (sh, 4.43), 310 (sh, 4.29), 327 (4.44), 344 (4.39)
2-Propanol	256 (4.49), 268 (4.52), 288 (sh, 4.32), 309 (sh, 4.21), 324 (4.32), 340 (4.27), 370 (sh, 3.45)
Ethanol	256 (4.48), 268 (4.50), 286 (sh, 4.33), 308 (sh, 4.22), 324 (4.34), 339 (4.29)
Acetonitrile	256 (4.52), 268 (4.52), 287 (sh, 4.33), 308 (sh, 4.20), 325 (4.34), 341 (4.31), 369 (sh, 3.68)
DMSO	258 (4.62), 271 (4.60), 289 (sh, 4.42), 314 (sh, 4.25), 330 (4.34), 348 (4.29), 377 (sh, 3.43)

Compound **1g**.

**Solvent**	**λ_{max} (log ε) nm**
Cyclohexane	259 (4.88), 287 (sh, 4.64), 342 (4.70), 366 (4.67)
Benzene	276 (4.68), 289 (sh, 4.61), 352 (4.64)
Dichloromethane	262 (4.80), 290 (sh, 4.54), 357 (4.62)
THF	260 (4.87), 289 (sh, 4.60), 355 (4.65)
2-Propanol	259 (4.80), 287 (sh, 4.56), 349 (4.62)
Ethanol	259 (4.84), 288 (sh, 4.59), 349 (4.64)
Acetonitrile	260 (4.76), 287 (sh, 4.51), 356 (4.59)
DMSO	264 (4.88), 292 (sh, 4.54), 366 (4.58)

CV measurements.

CV of all the compounds was recorded in acetonitrile at 1mM concentration at a scan rate of 0.1 V/s. using tetrabutylammonium tetrafluoroborate (0.1 M) as the supporting electrolyte, glassy carbon as the working electrode, Pt wire as the counter electrode and SCE as the reference electrode with CH Instrument 660A. The peak potentials are reported with reference to SCE.

Table 2: Oxidation and reduction peak potentials of compounds **1a-g**.

Compound	$E_{p, ox}$ (V)	$E_{p, red}$ (V)
**1a**	1.60	-2.14, -2.25
**1b**	1.65	-2.02, -2.15, -2.42
**1c**	1.68	-1.85, -2.03, -2.43, -2.55
**1d**	1.66	-1.72, -1.85, -2.09
**1e**	1.63	-1.63, -1.84, -2.18, -2.53
**1g**	0.76, 1.64	-2.27, -2.36, -2.53

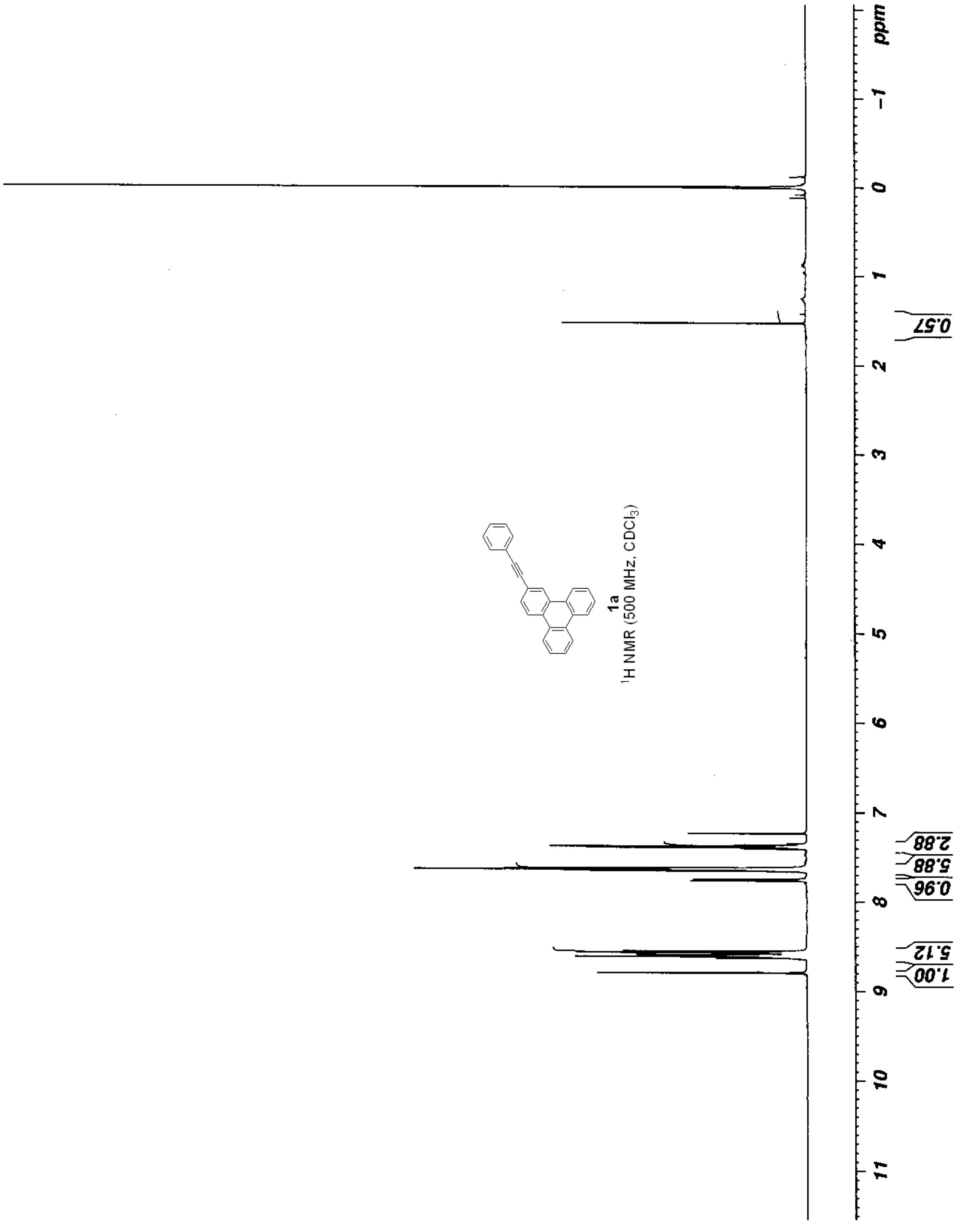

Figure 1: ^{1}H NMR spectrum of **1a** in $CDCl_3$.

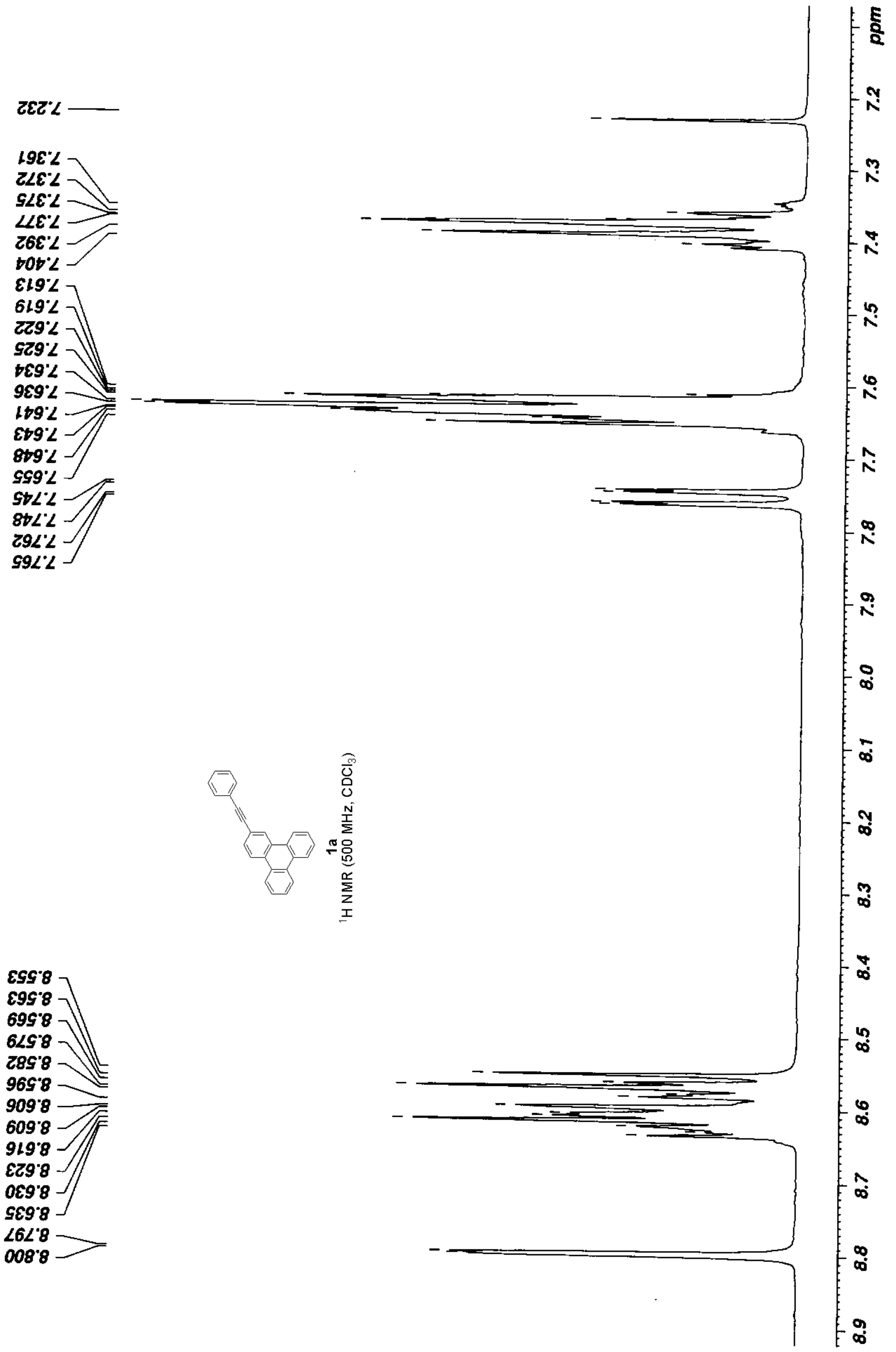

Figure 2: Expanded aromatic region of ^{1}H NMR spectrum of **1a** in $CDCl_3$.

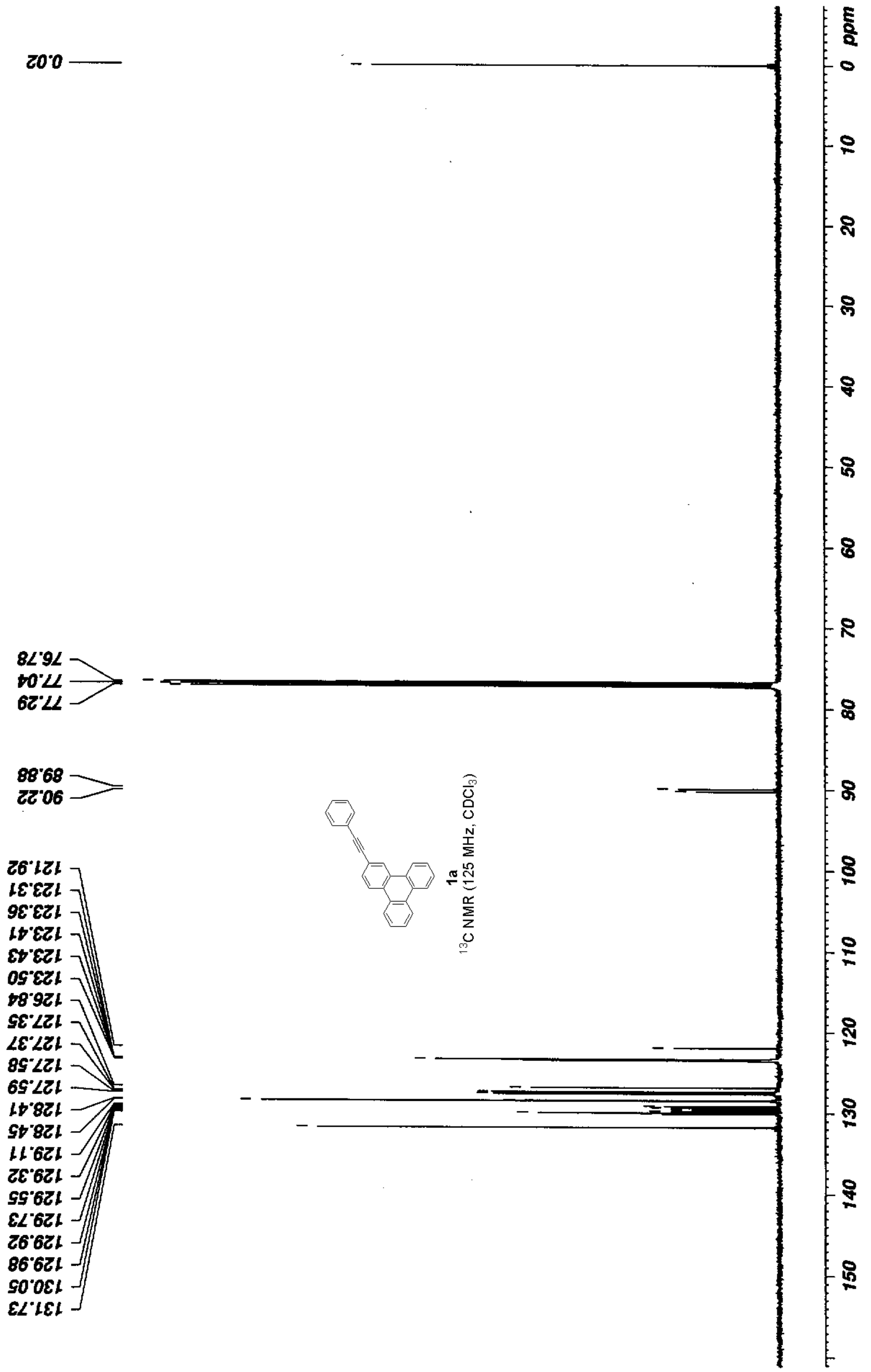

Figure 3: ^{13}C NMR spectrum of **1a** in $CDCl_3$.

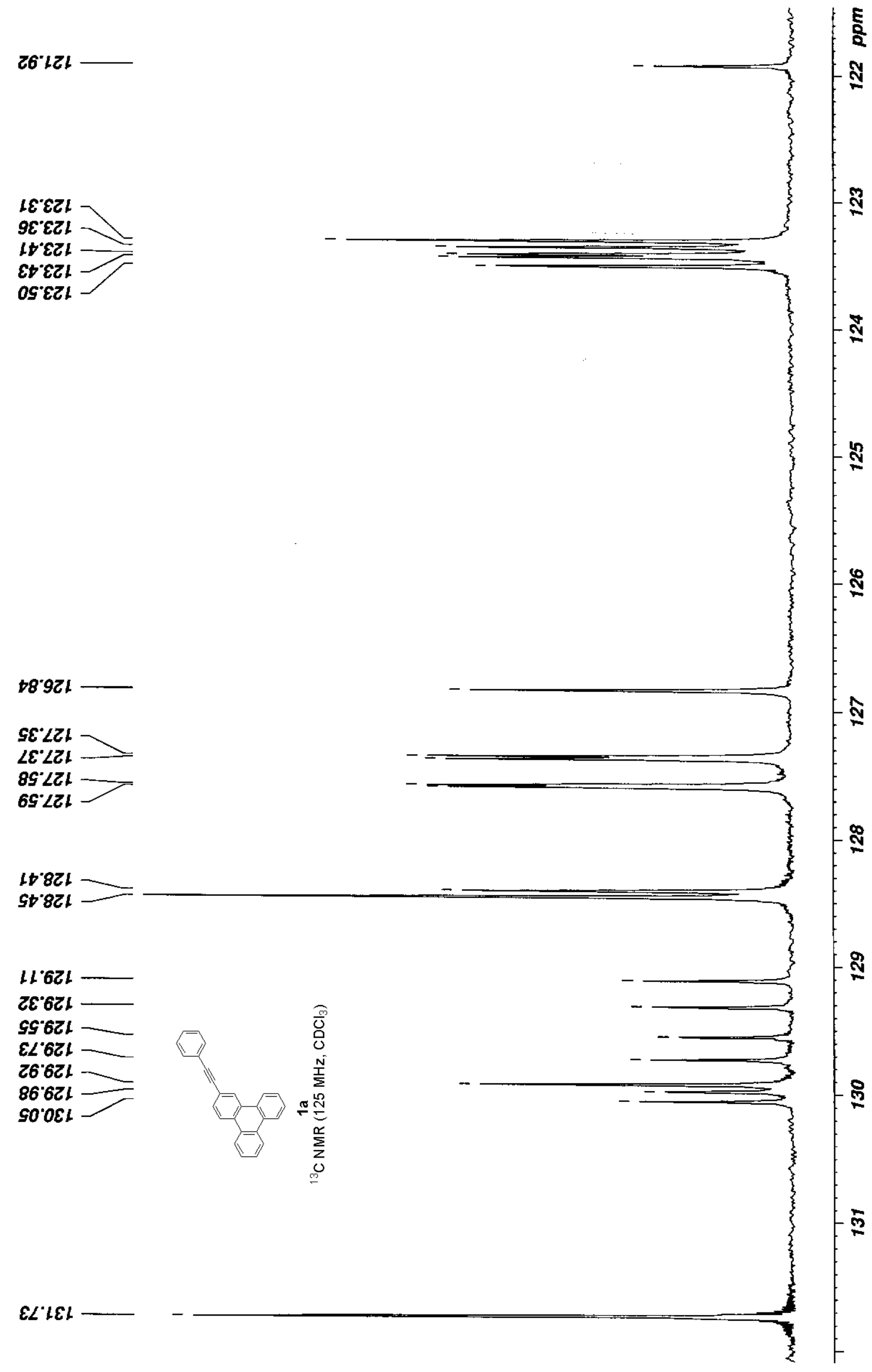

Figure 4: Expanded aromatic region of ^{13}C NMR spectrum of **1a** in $CDCl_3$.

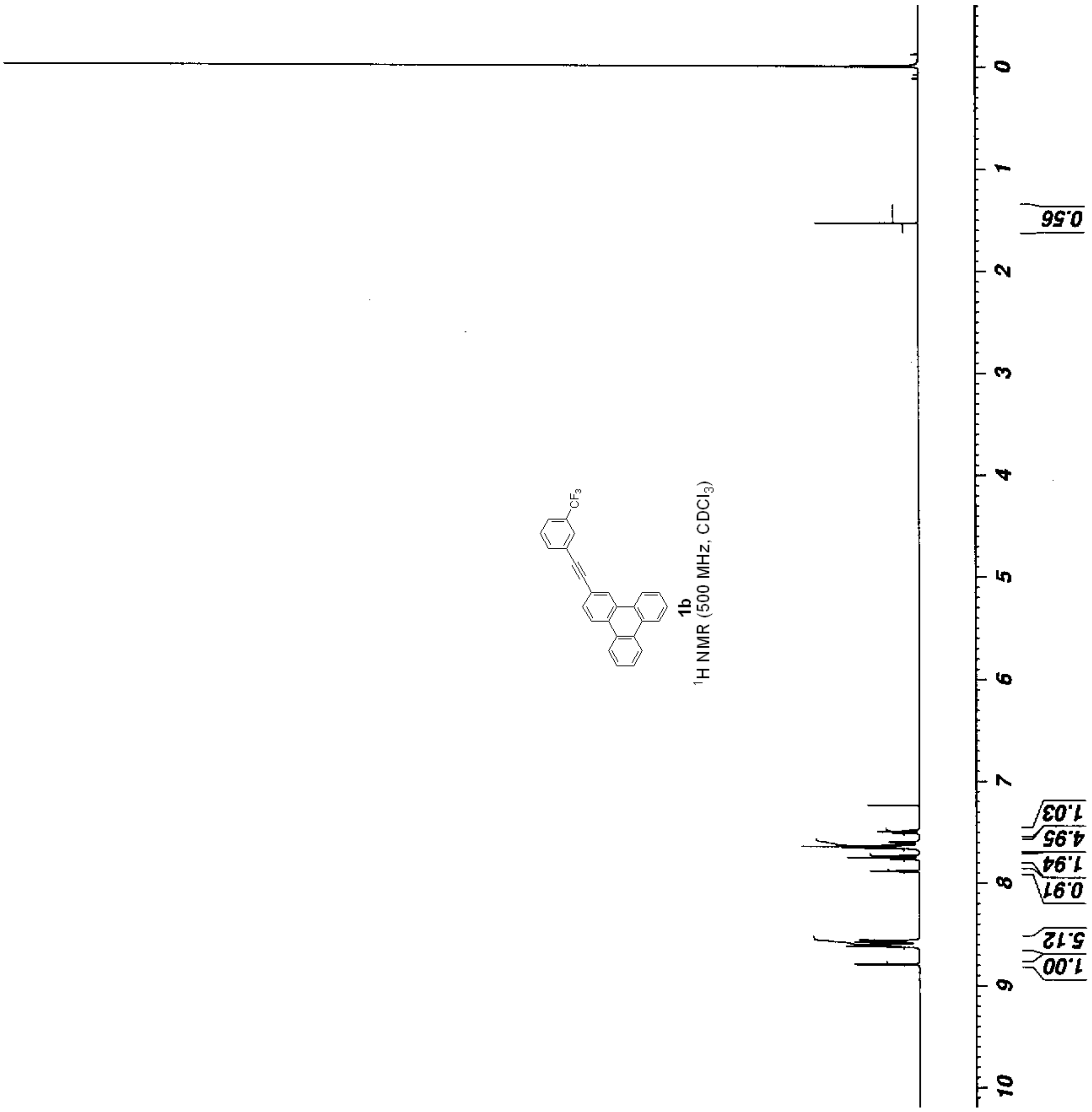

Figure 5: ^{1}H NMR spectrum of **1b** in $CDCl_3$.

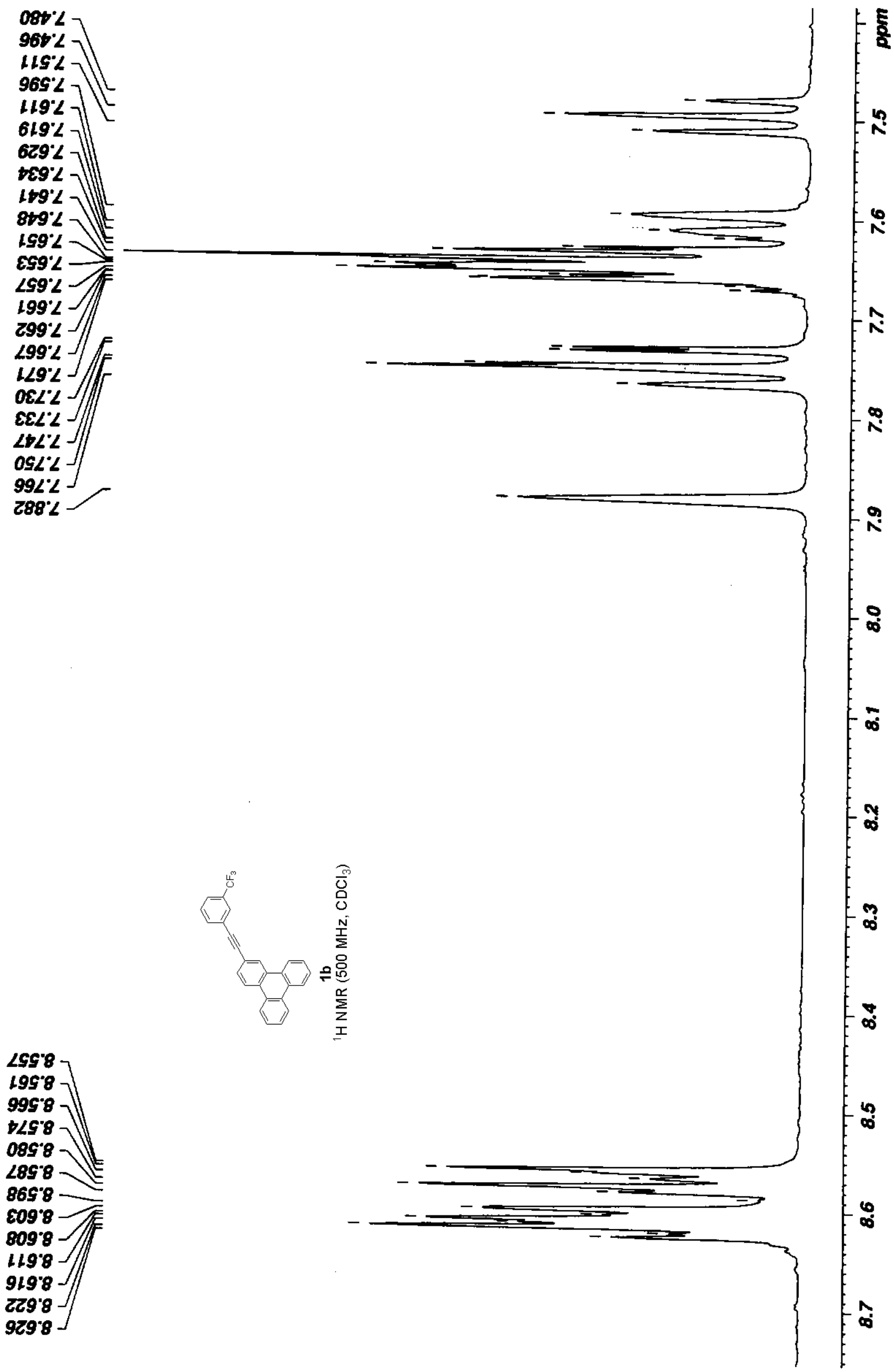

Figure 6: Expanded aromatic region of ^{1}H NMR spectrum of **1b** in $CDCl_3$.

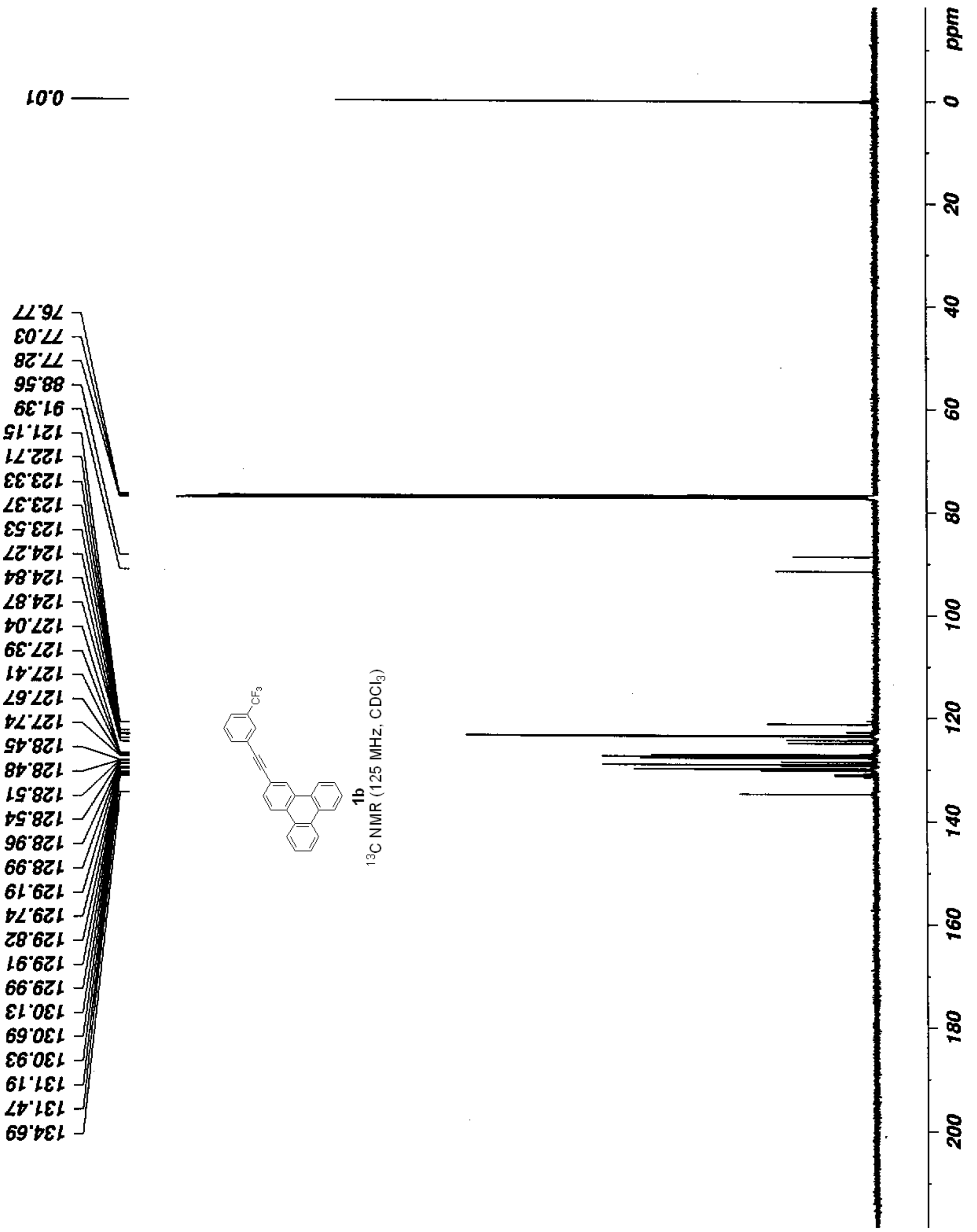

Figure 7: ^{13}C NMR spectrum of **1b** in $CDCl_3$.

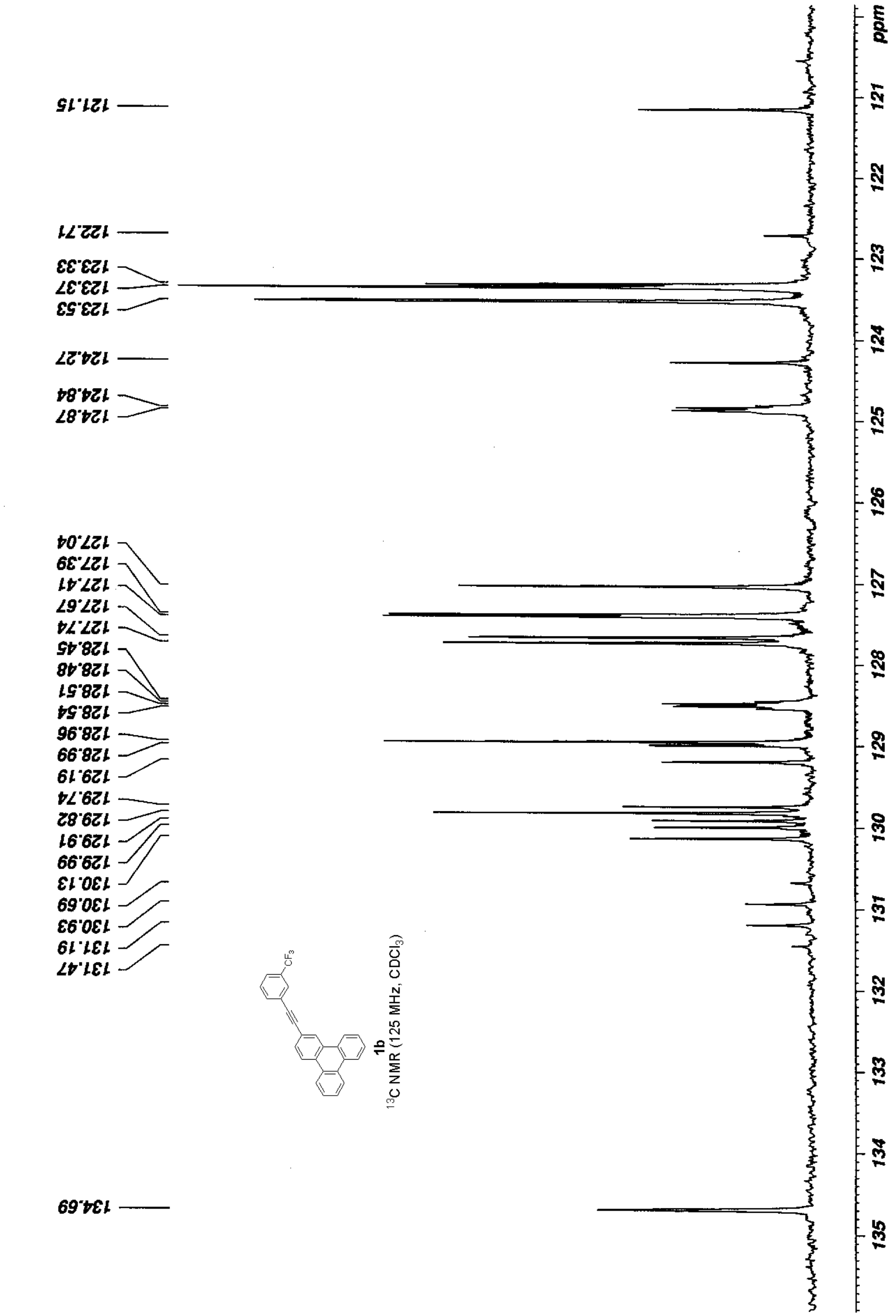

Figure 8: Expanded aromatic region of ^{13}C NMR spectrum of **1b** in $CDCl_3$.

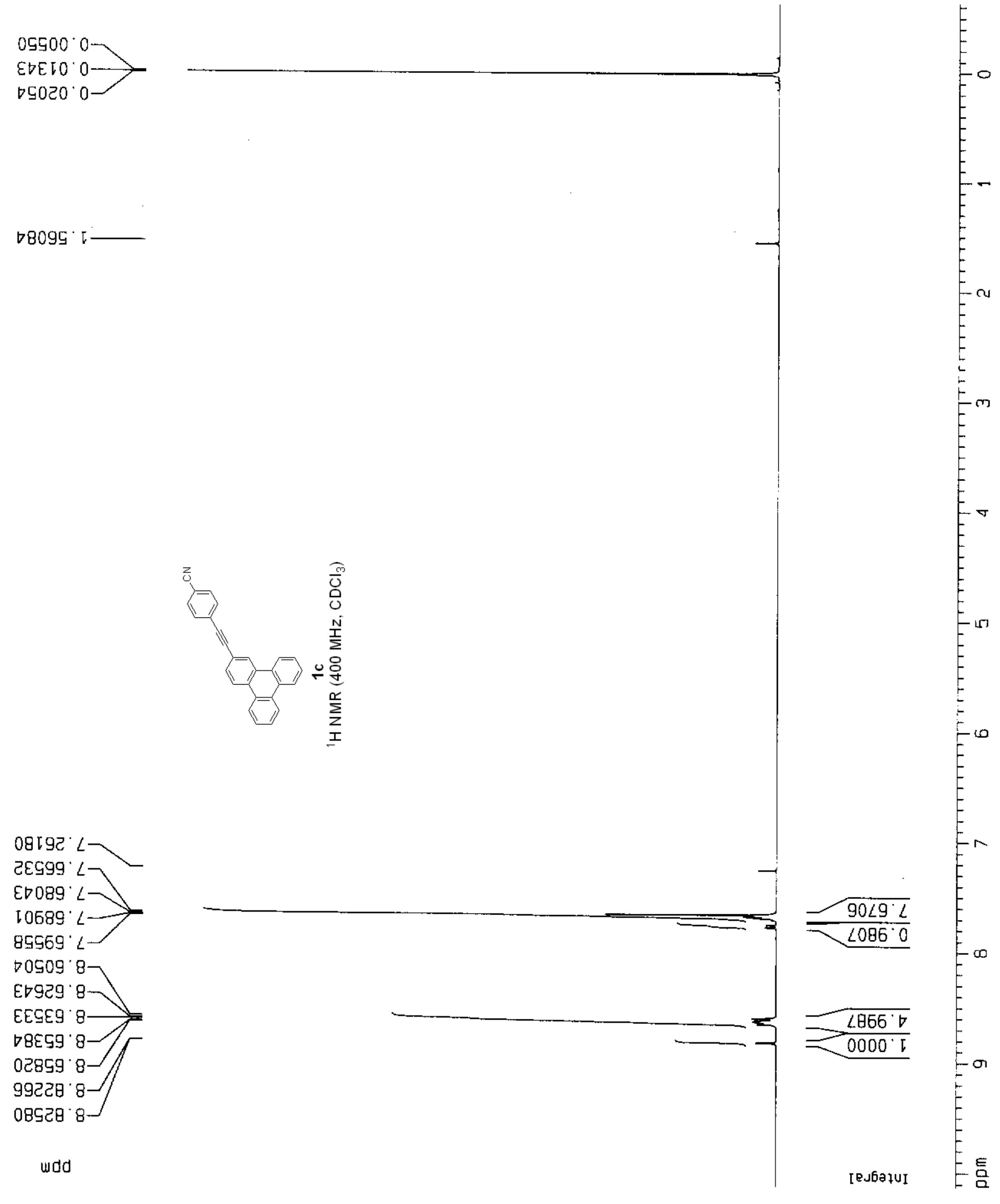

Figure 9: ^{1}H NMR spectrum of **1c** in $CDCl_3$.

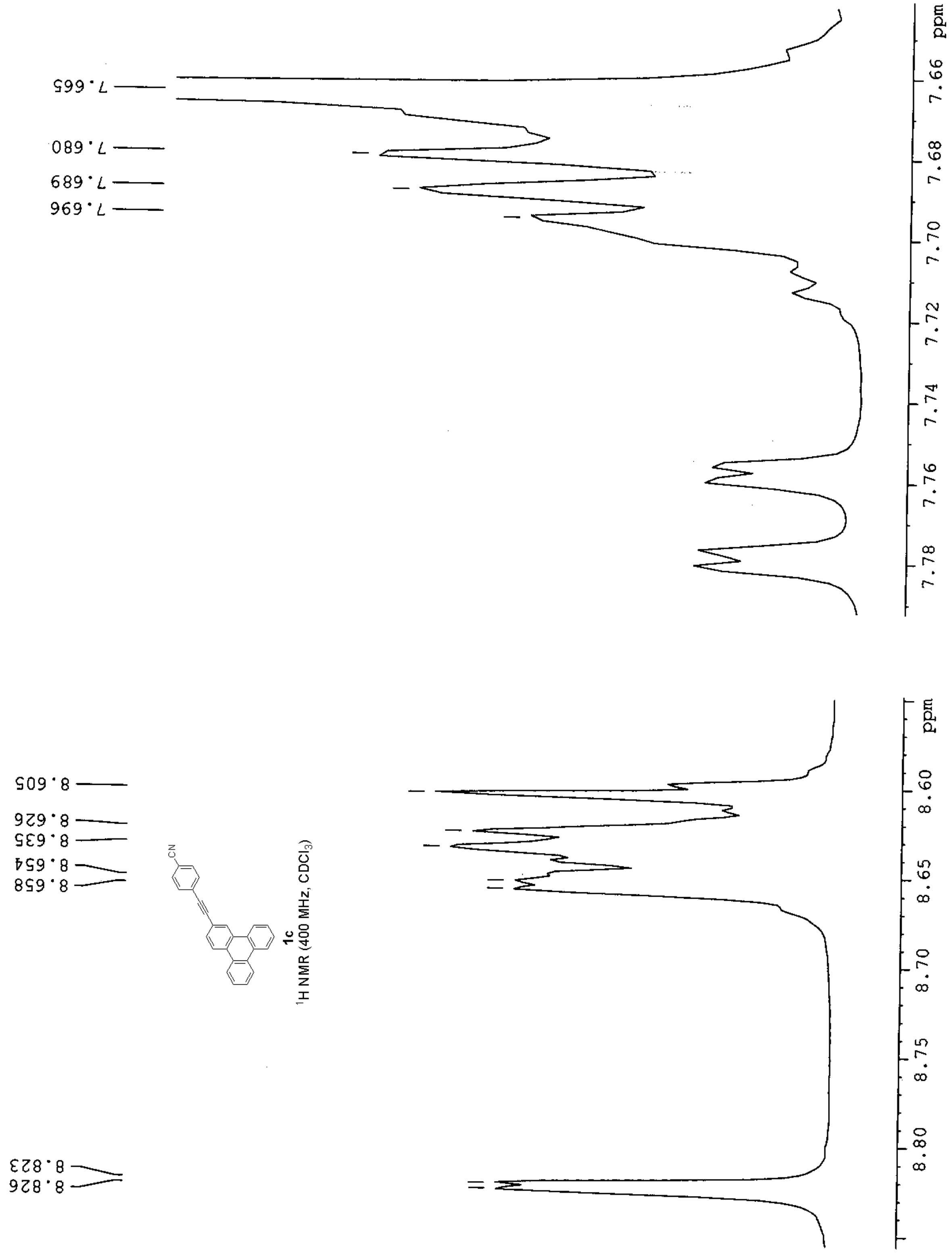

Figure 10: Expanded aromatic region of ^{1}H NMR spectrum of **1c** in $CDCl_3$.

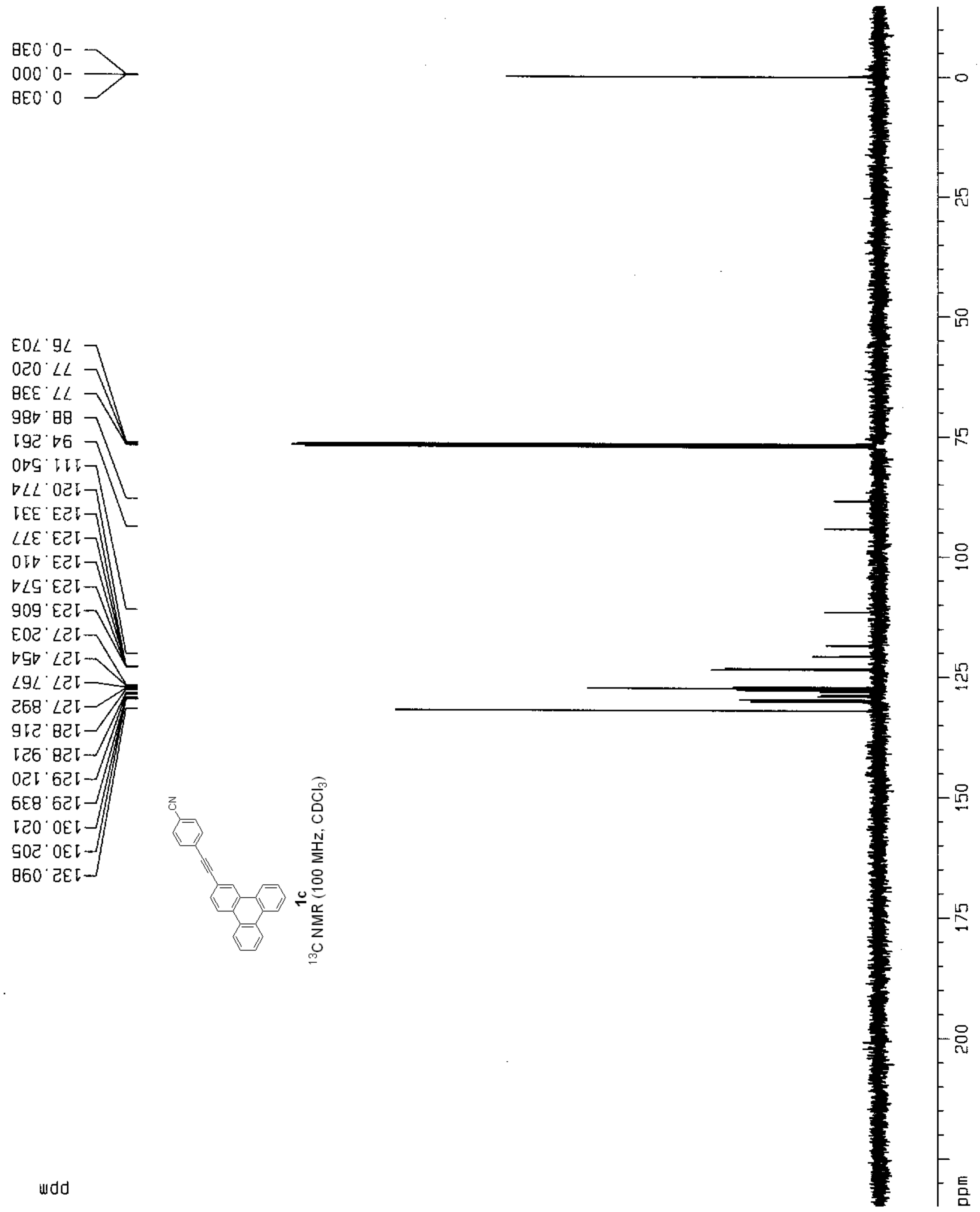

Figure 11: ^{13}C NMR spectrum of **1c** in $CDCl_3$.

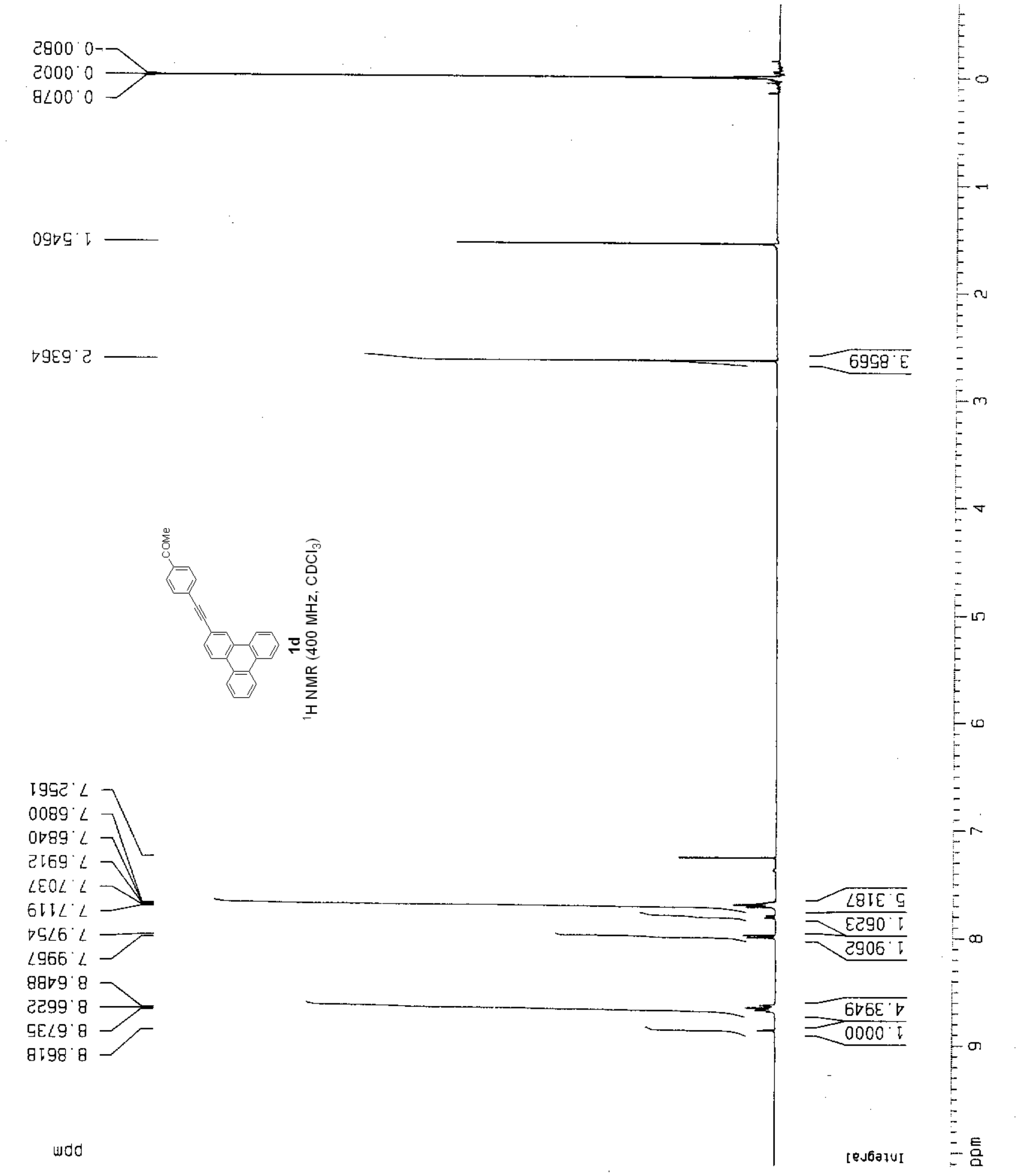

Figure 12: ^{1}H NMR spectrum of **1d** in $CDCl_3$.

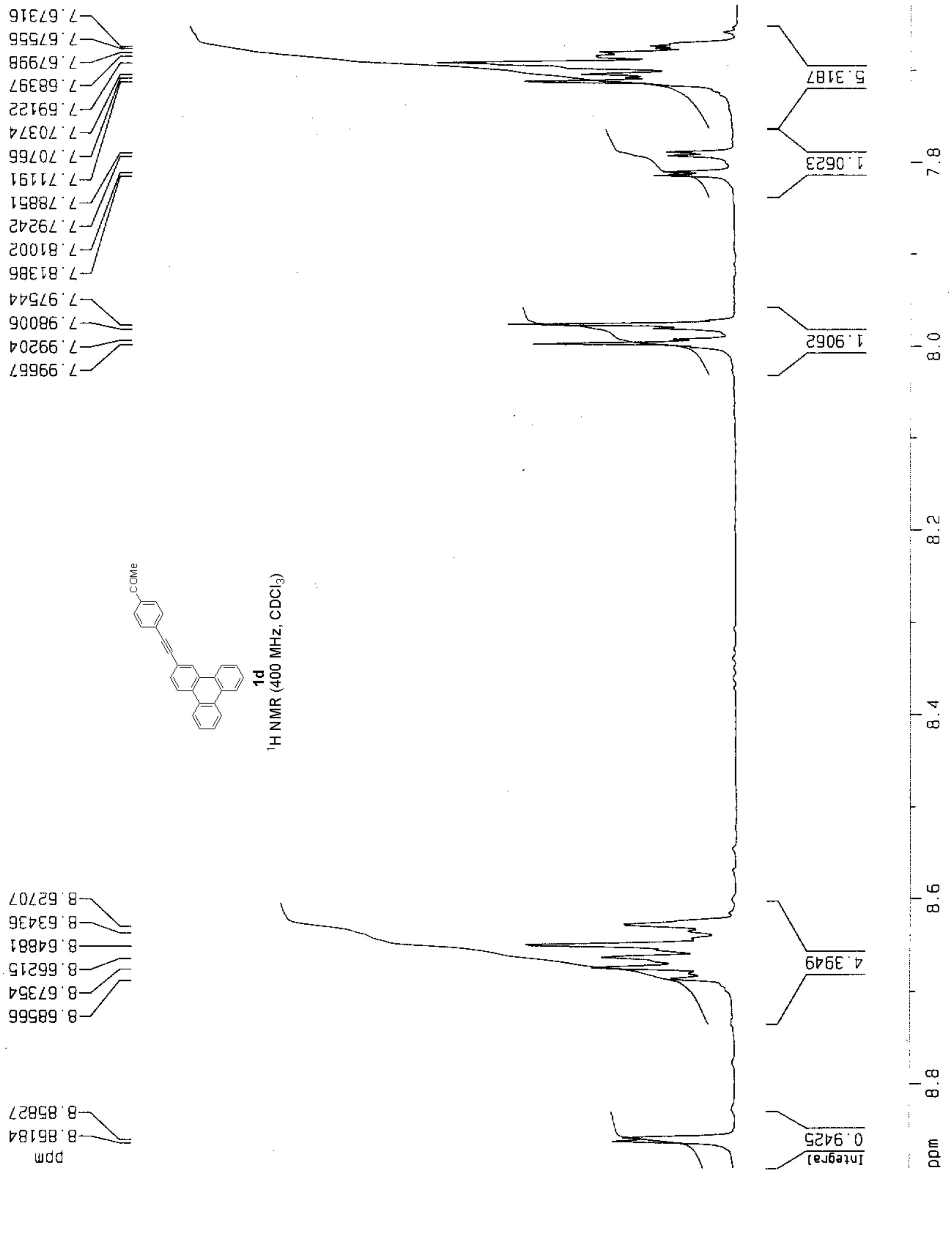

Figure 13: Expanded aromatic region of ^{1}H NMR spectrum of **1d** in $CDCl_3$.

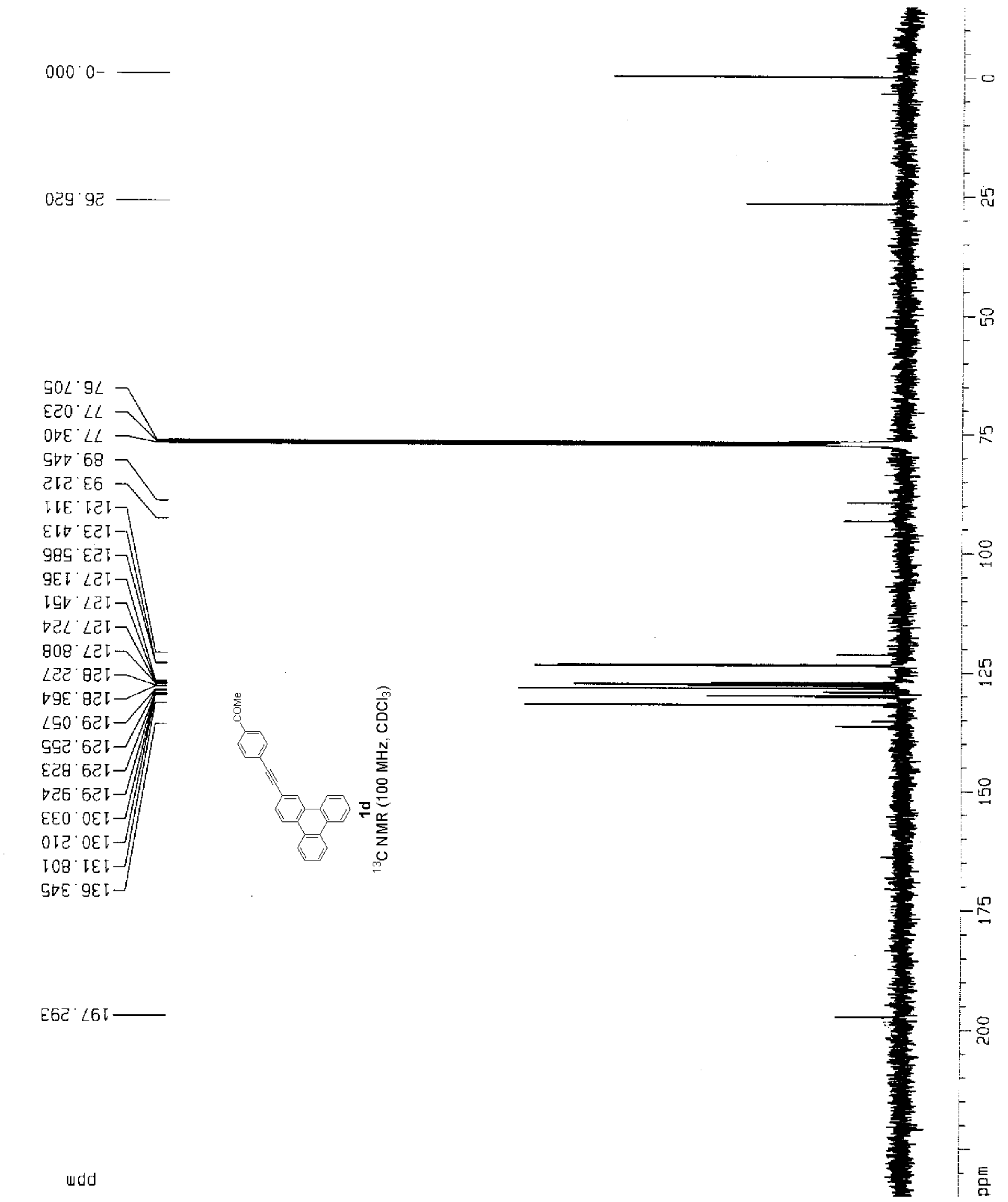

Figure 14: ^{13}C NMR spectrum of **1d** in $CDCl_3$.

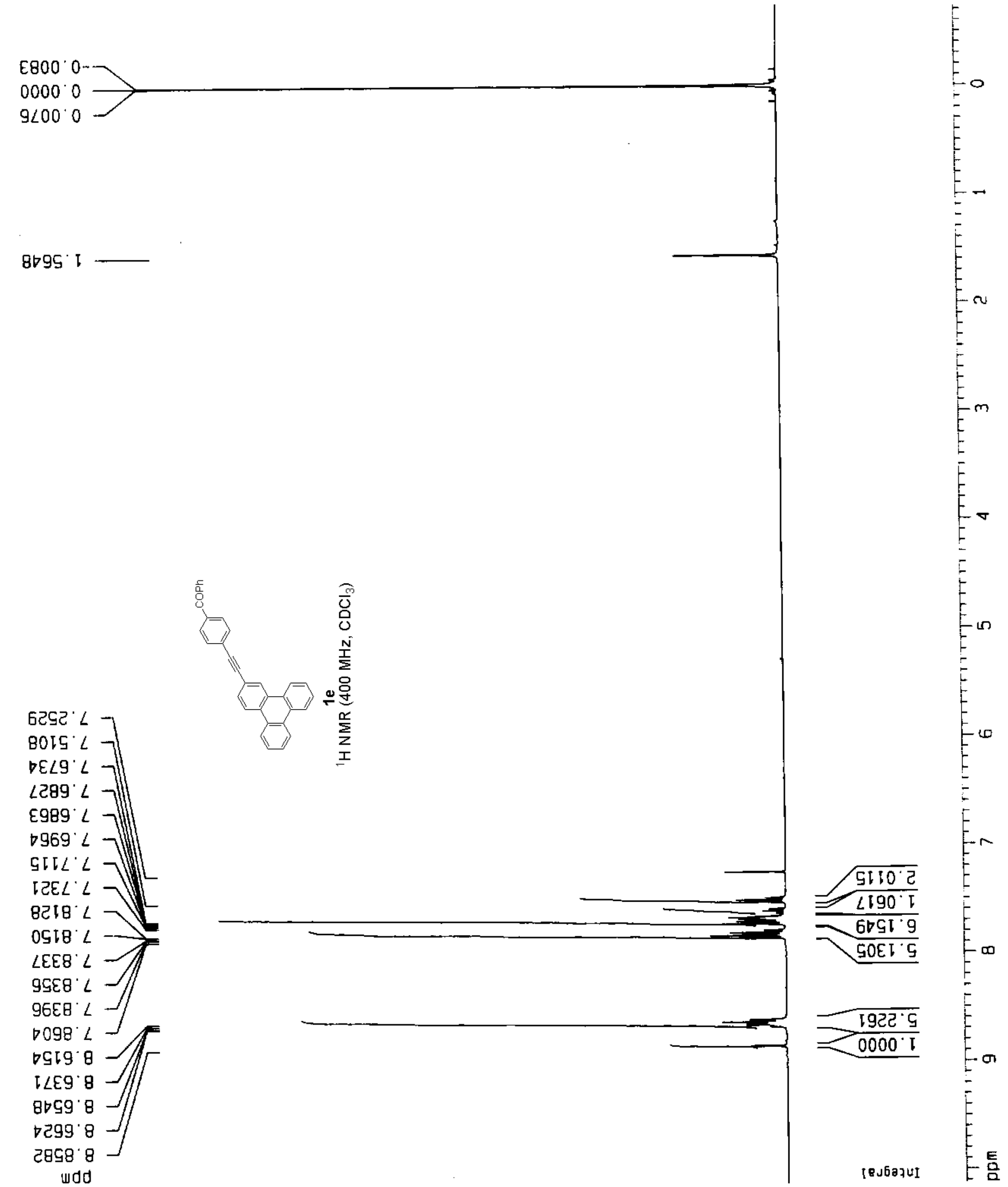

Figure 15: ^{1}H NMR spectrum of **1e** in $CDCl_3$.

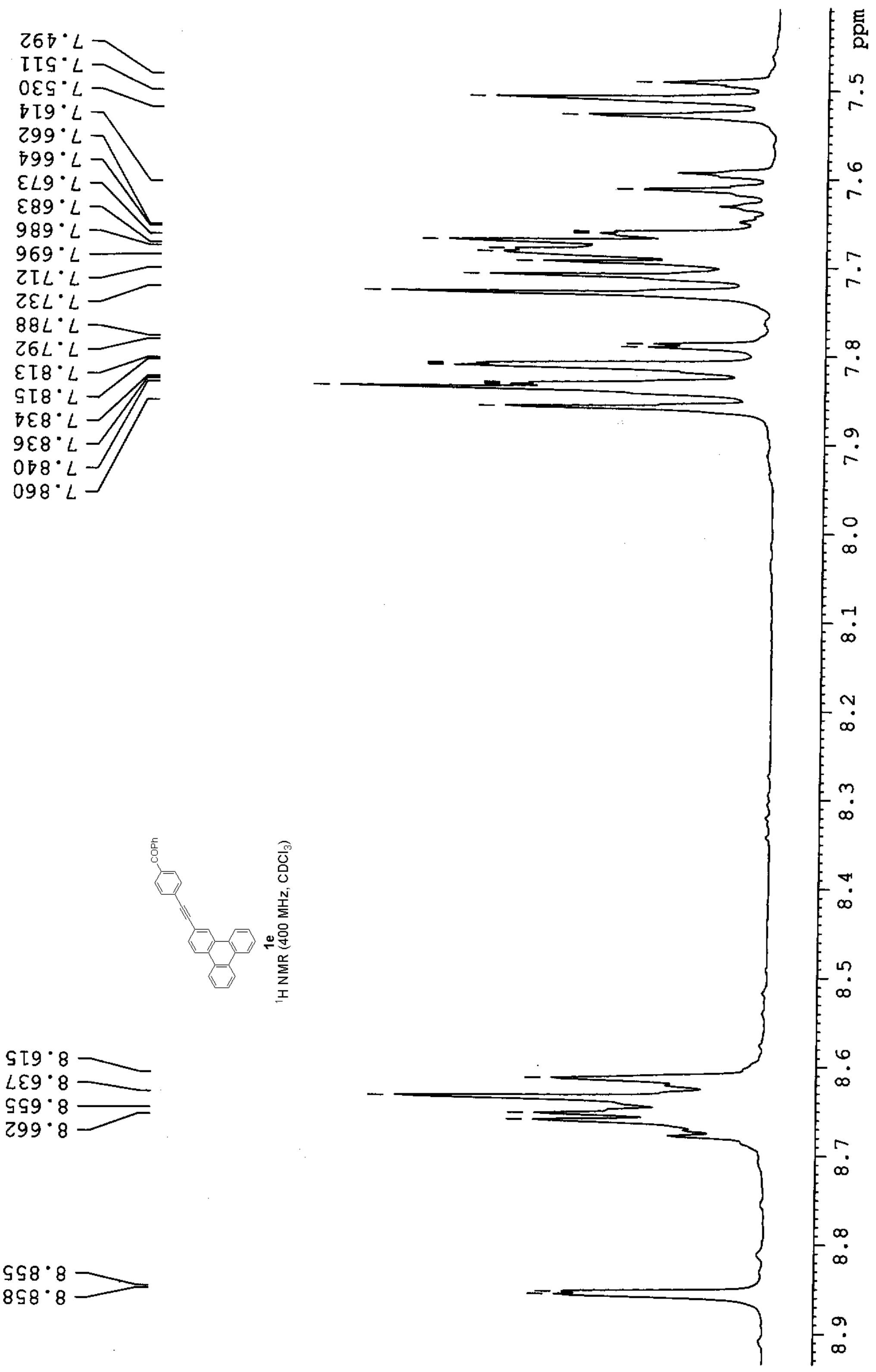

Figure 16: Expanded aromatic region of ^{1}H NMR spectrum of **1e** in $CDCl_3$.

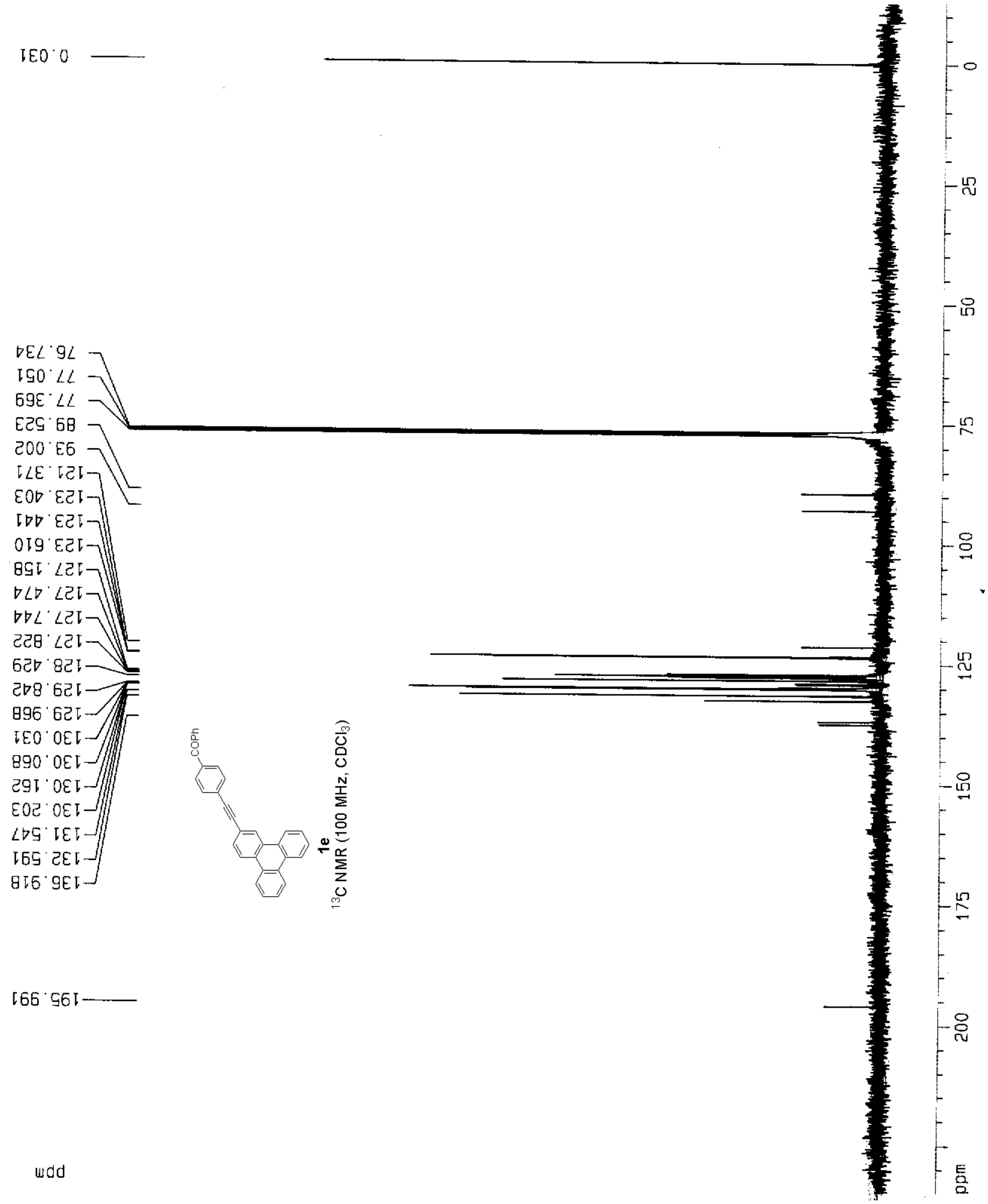

Figure 17: ^{13}C NMR spectrum of **1e** in $CDCl_3$.

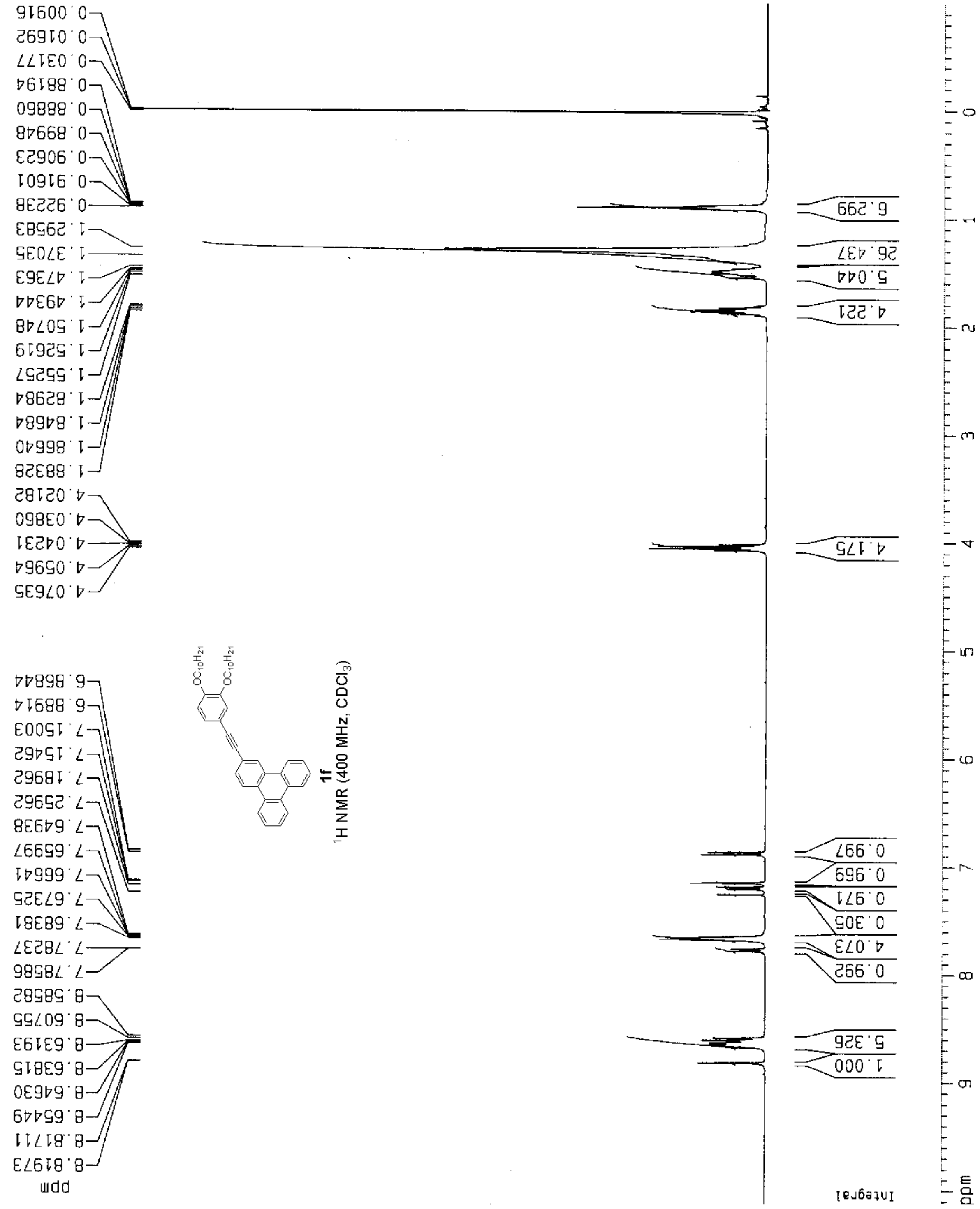

Figure 18: ^{1}H NMR spectrum of **1f** in $CDCl_3$.

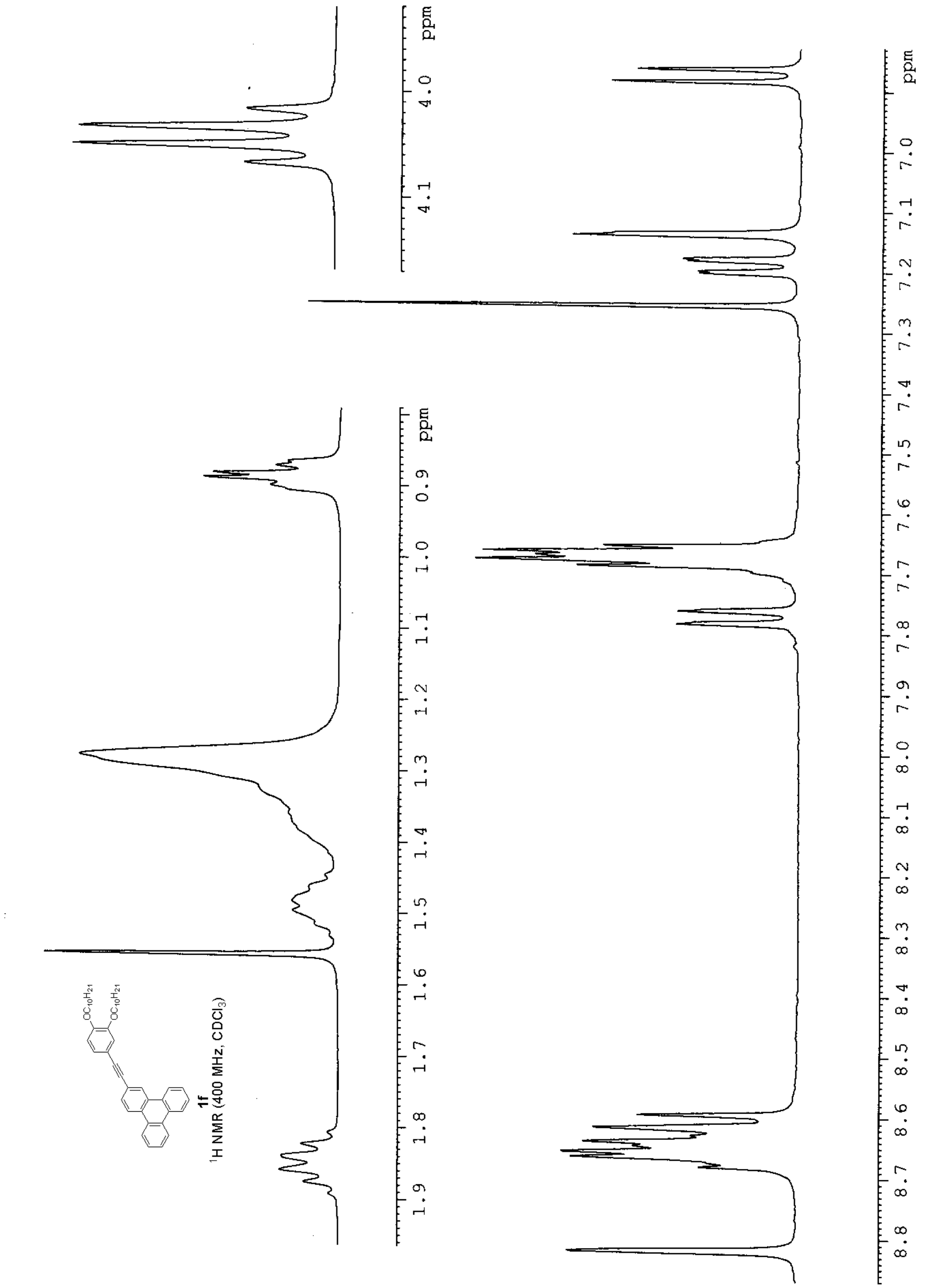

Figure 19: Expanded aromatic and aliphatic regions of ^{1}H NMR spectrum of **1f** in $CDCl_3$.

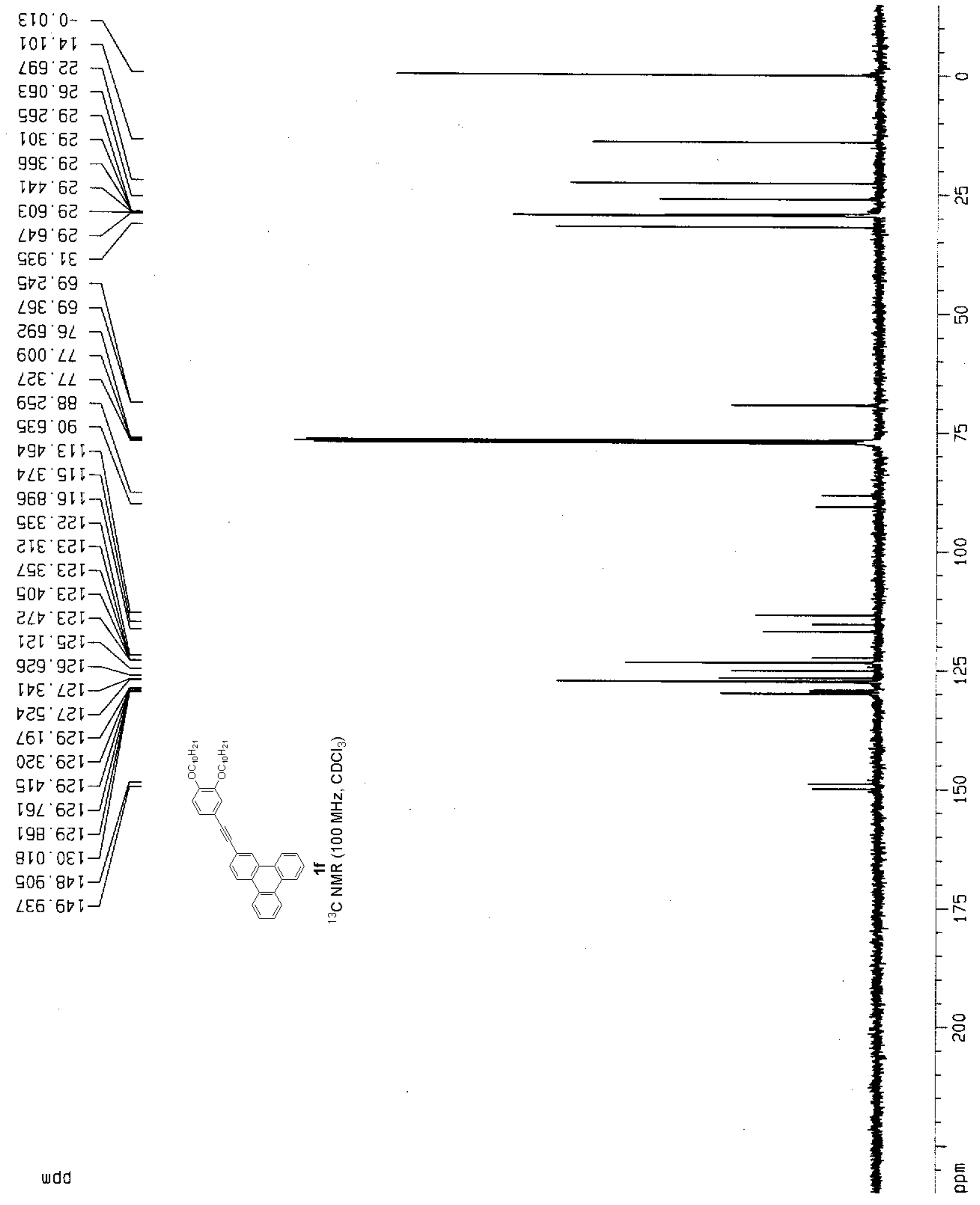

Figure 20: ^{13}C NMR spectrum of **1f** in $CDCl_3$.

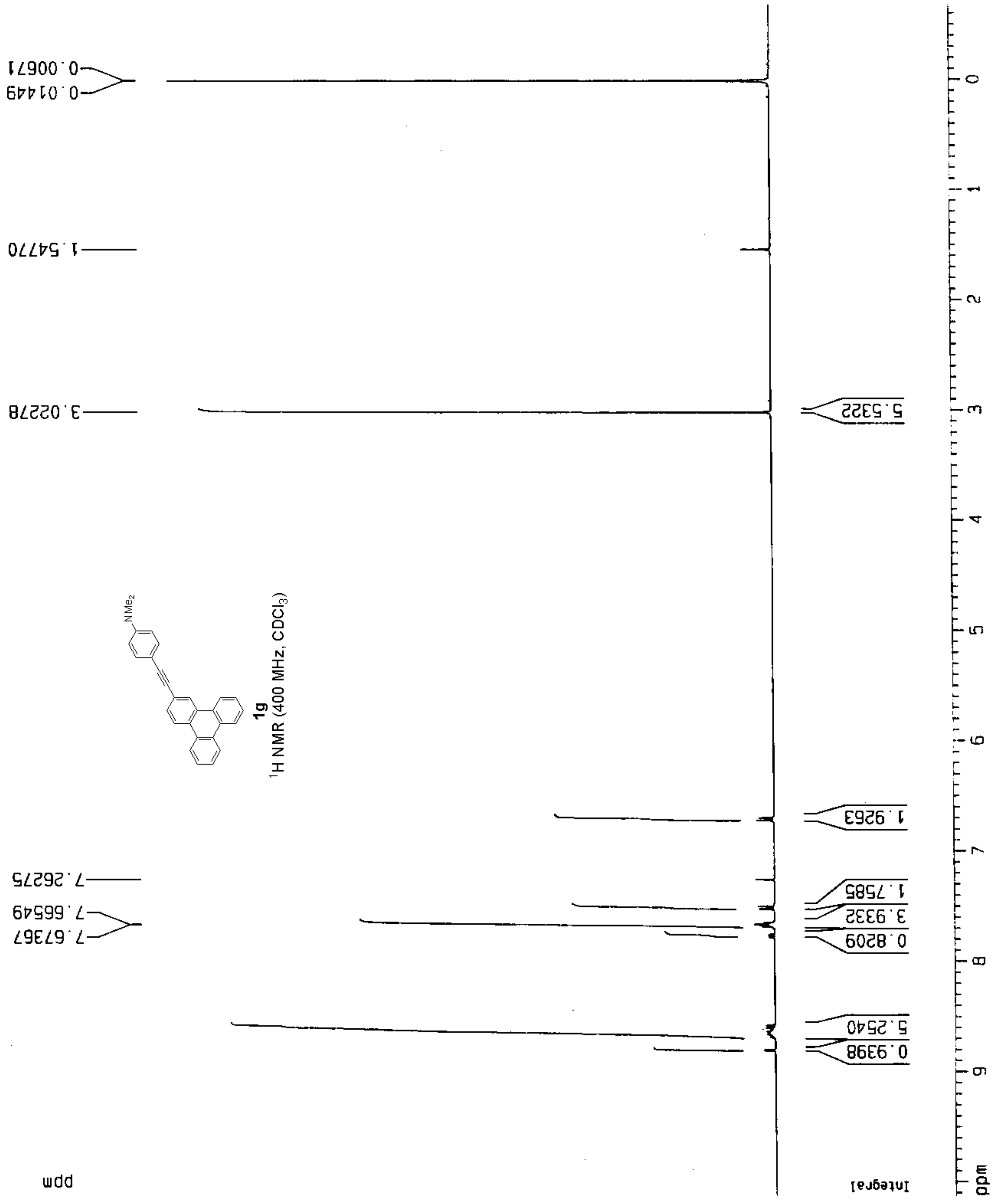

Figure 21: ^{1}H NMR spectrum of **1g** in $CDCl_3$.

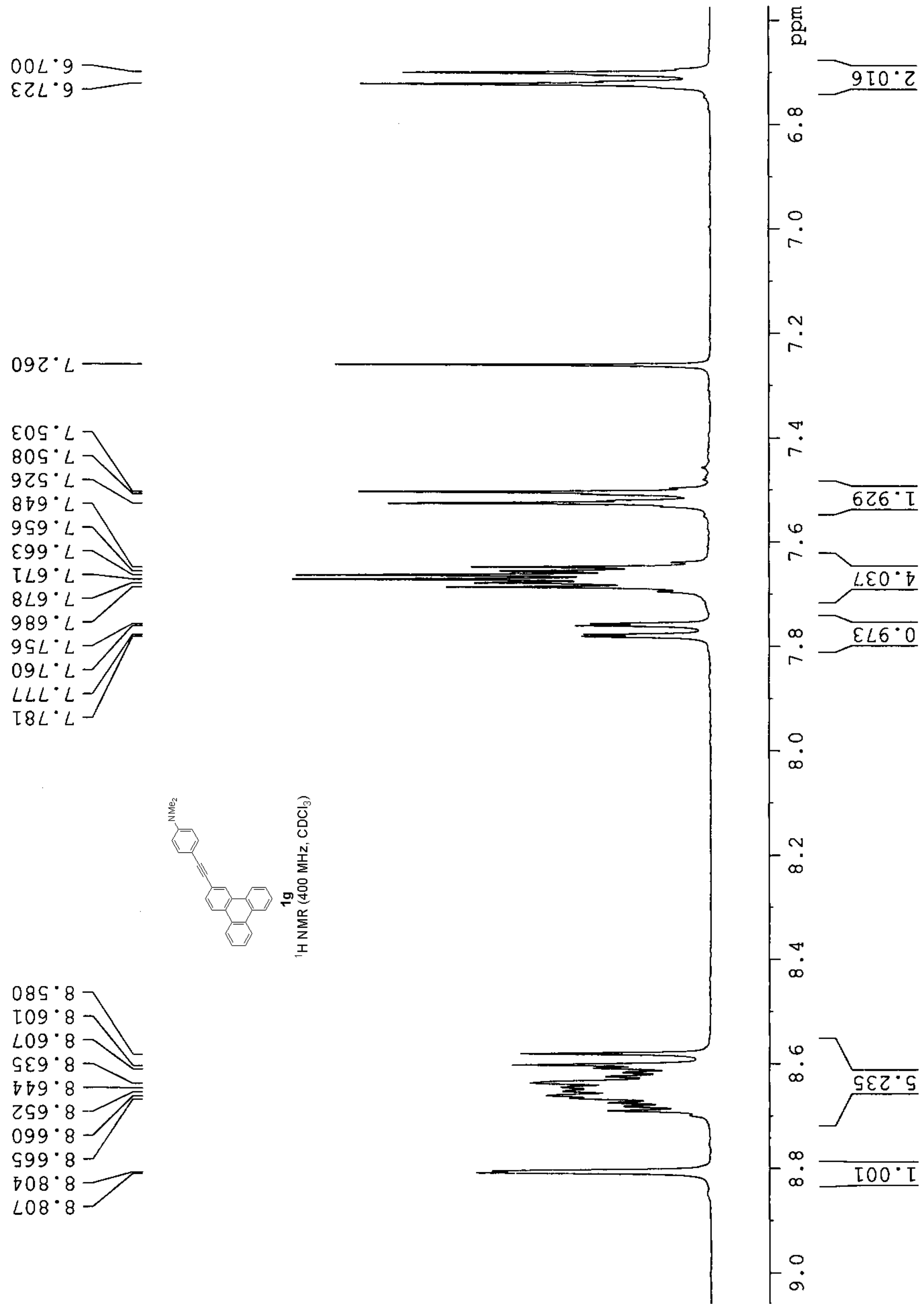

Figure 22: Expanded aromatic region of ^{1}H NMR spectrum of **1g** in $CDCl_3$.

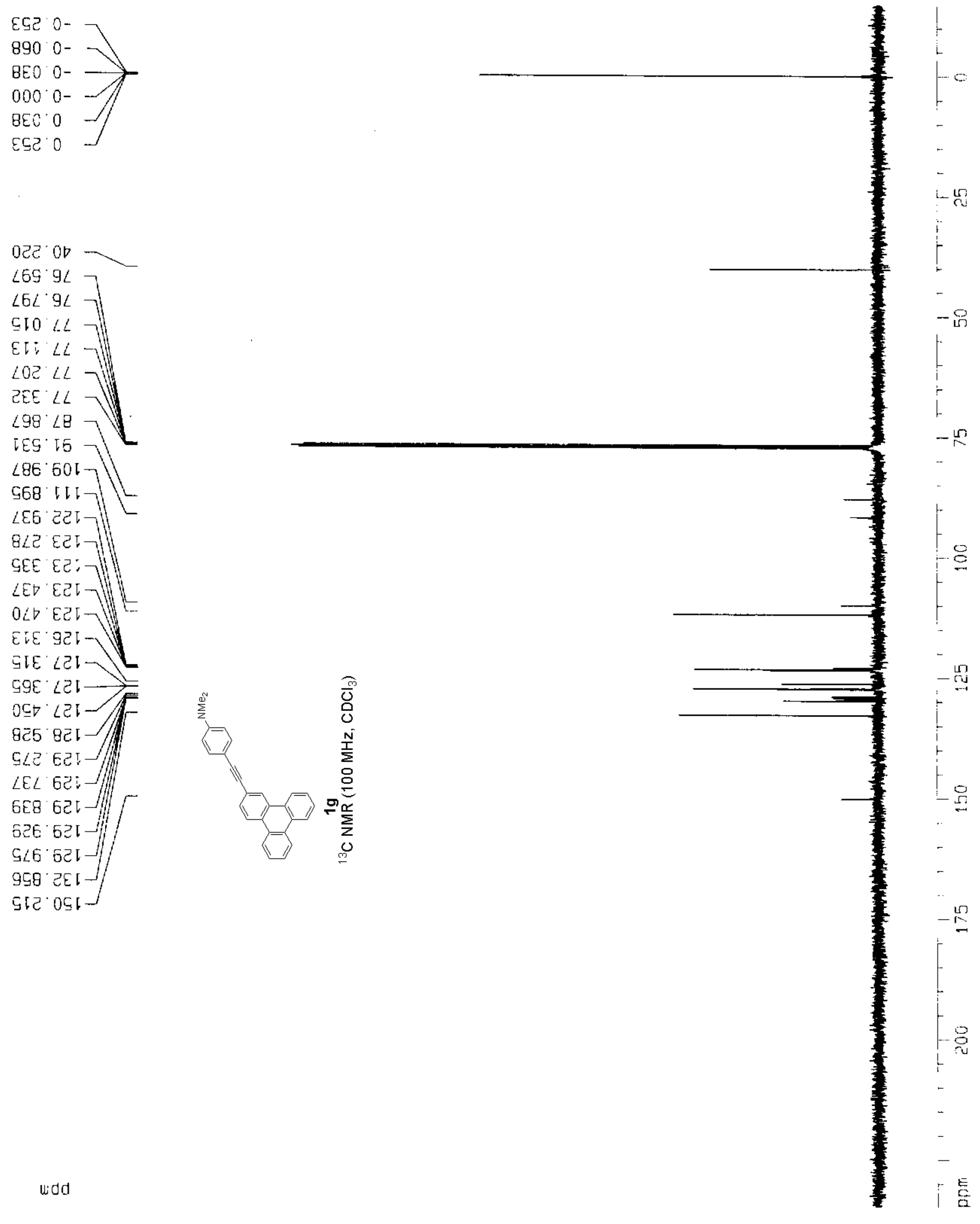

Figure 23: ^{13}C NMR spectrum of **1g** in $CDCl_3$.

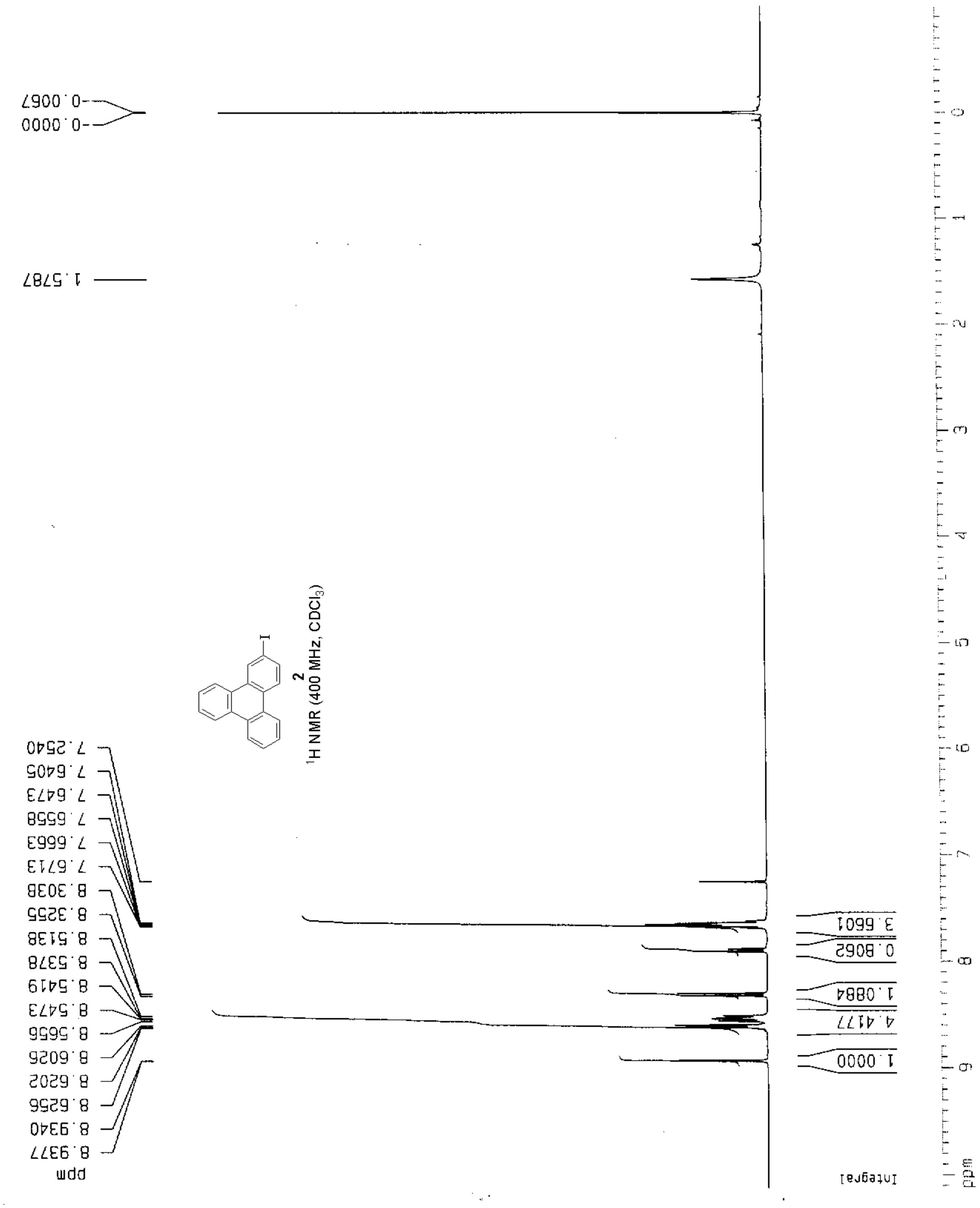

Figure 24: ^{1}H NMR spectrum of **2** in $CDCl_3$.

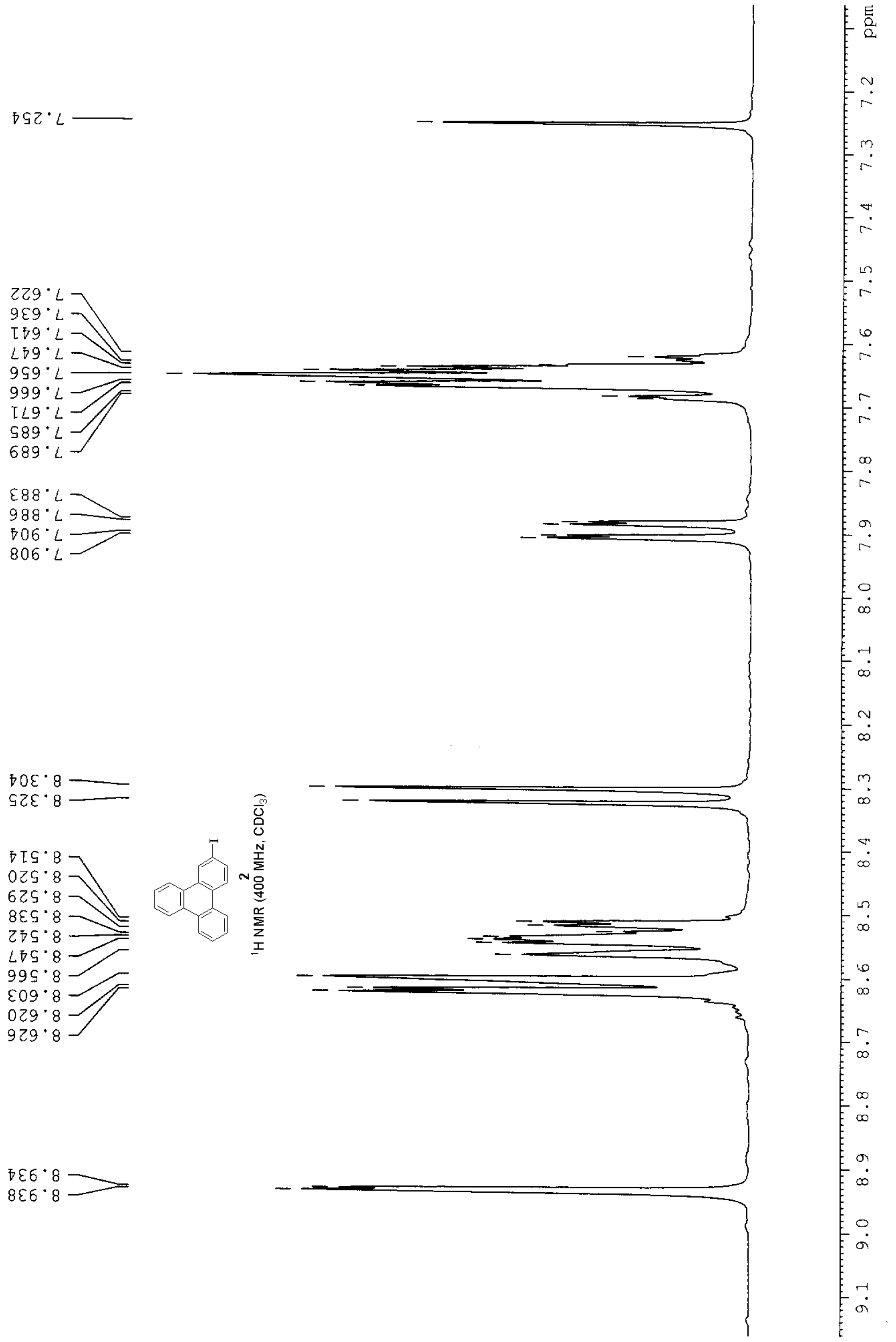

Figure 25: Expanded aromatic region of ^{1}H NMR spectrum of **2** in $CDCl_3$.

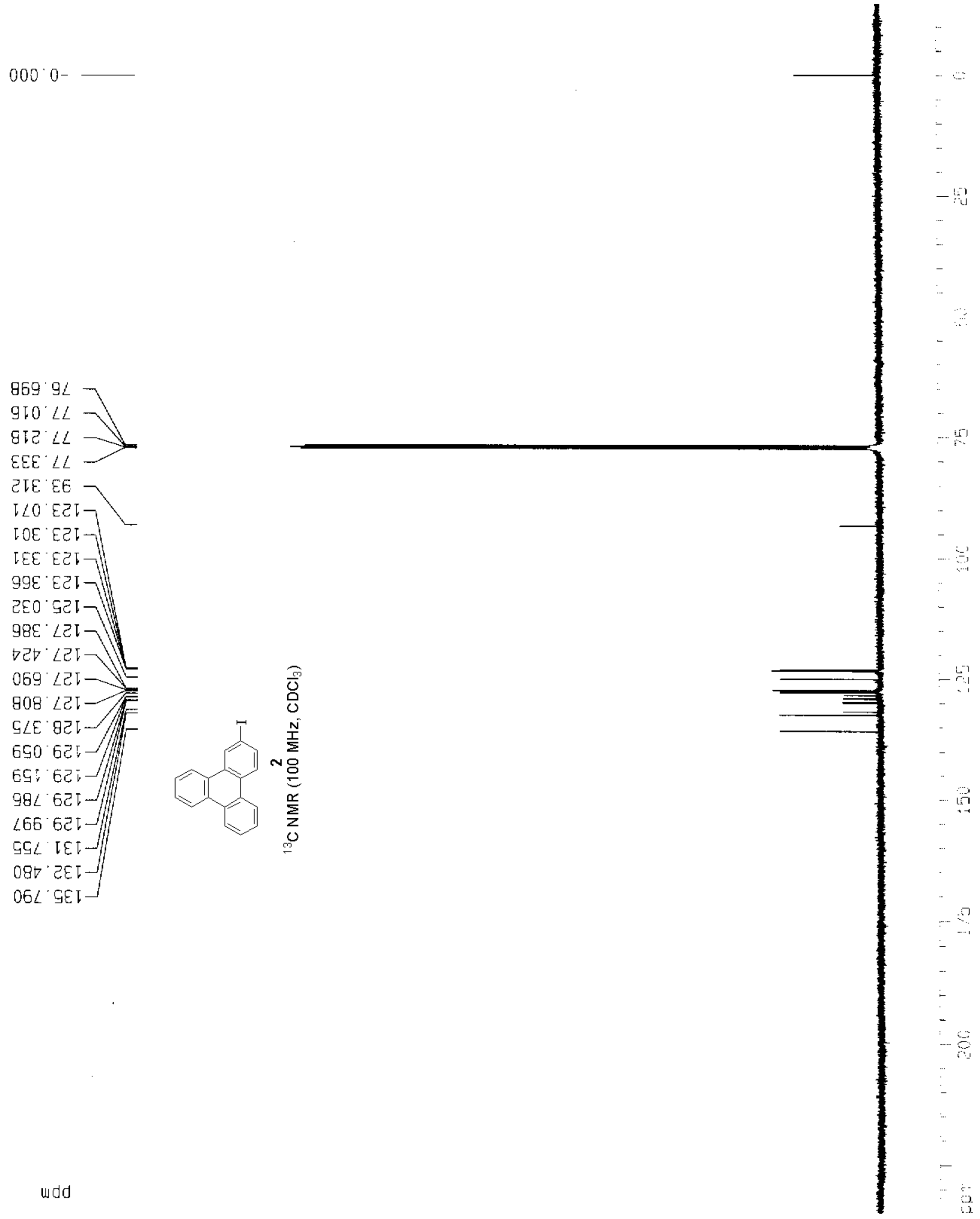

Figure 26: ^{13}C NMR spectrum of **2** in $CDCl_3$.

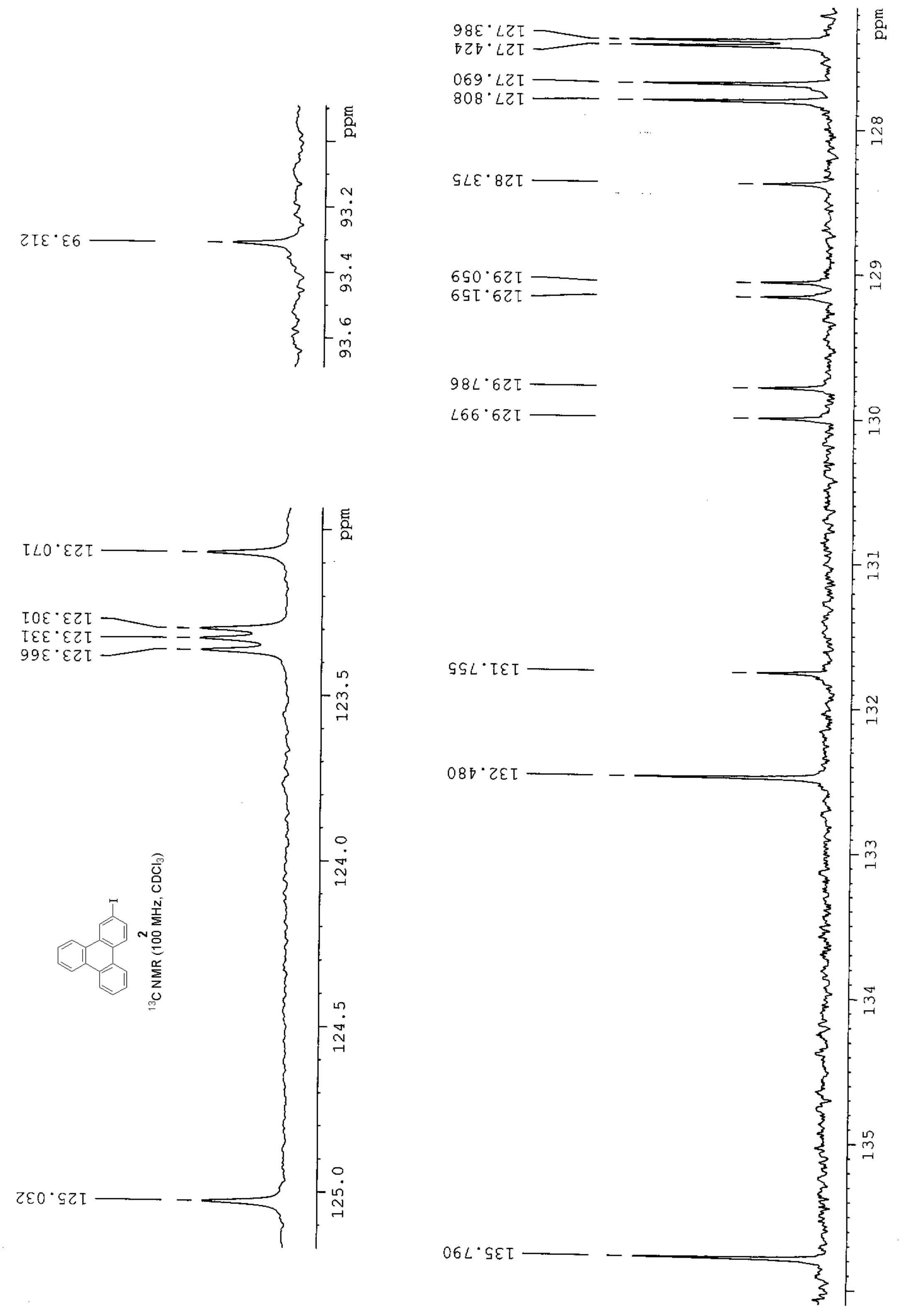

Figure 27: Expanded aromatic region of ^{13}C NMR spectrum of **2** in $CDCl_3$.

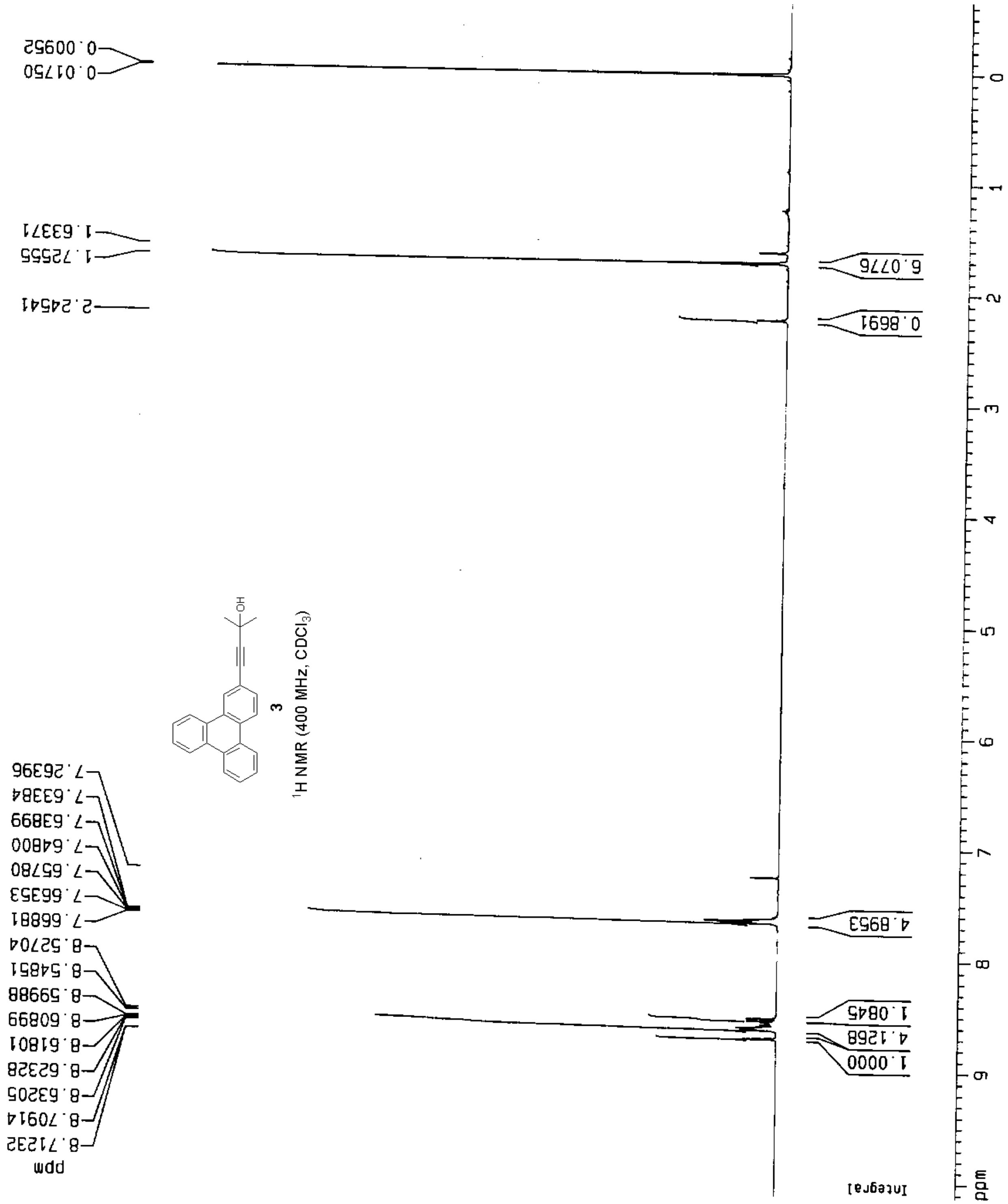

Figure 28: ^{1}H NMR spectrum of **3** in $CDCl_3$.

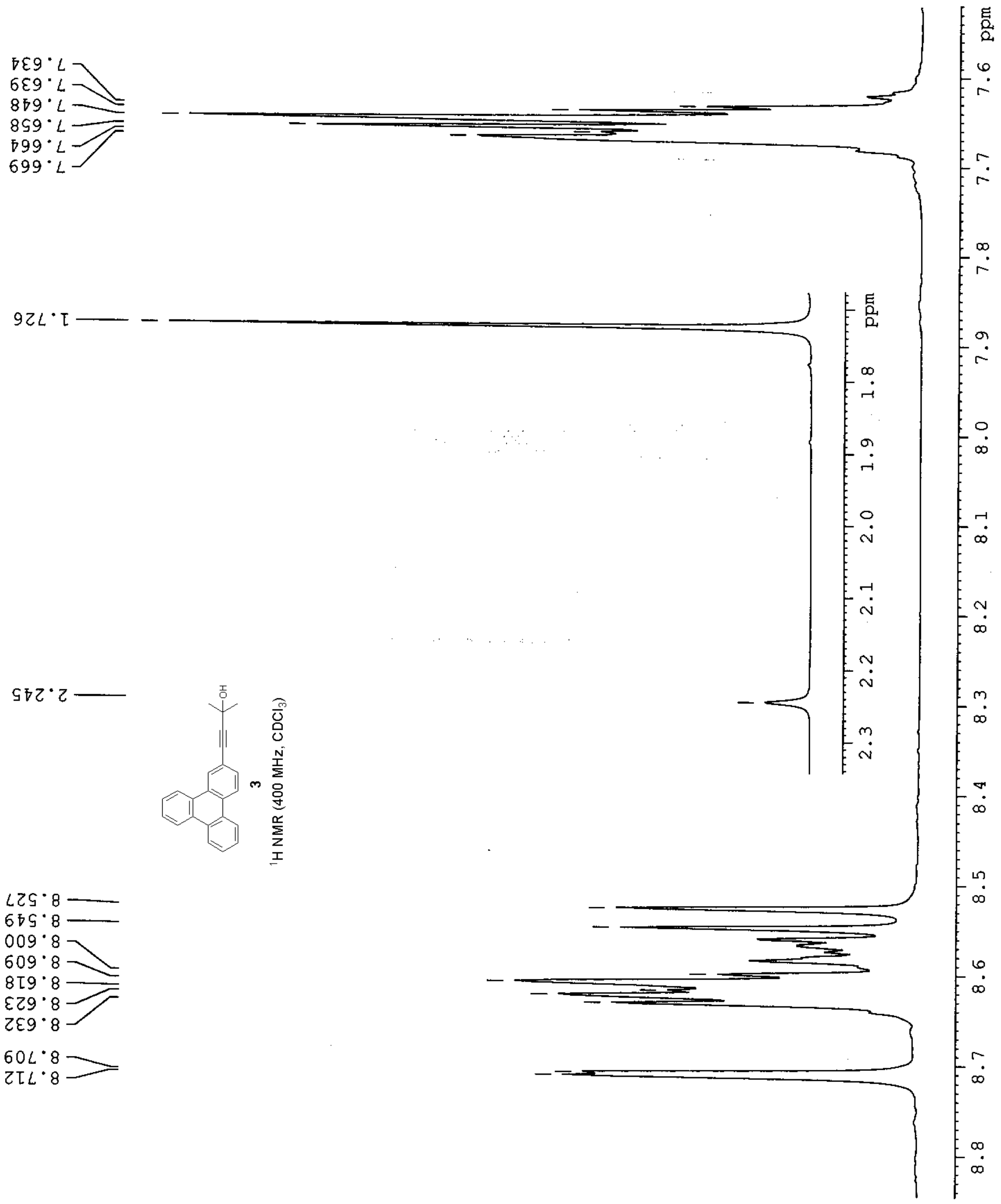

Figure 29: Expanded aromatic region of ^{1}H NMR spectrum of **3** in $CDCl_3$.

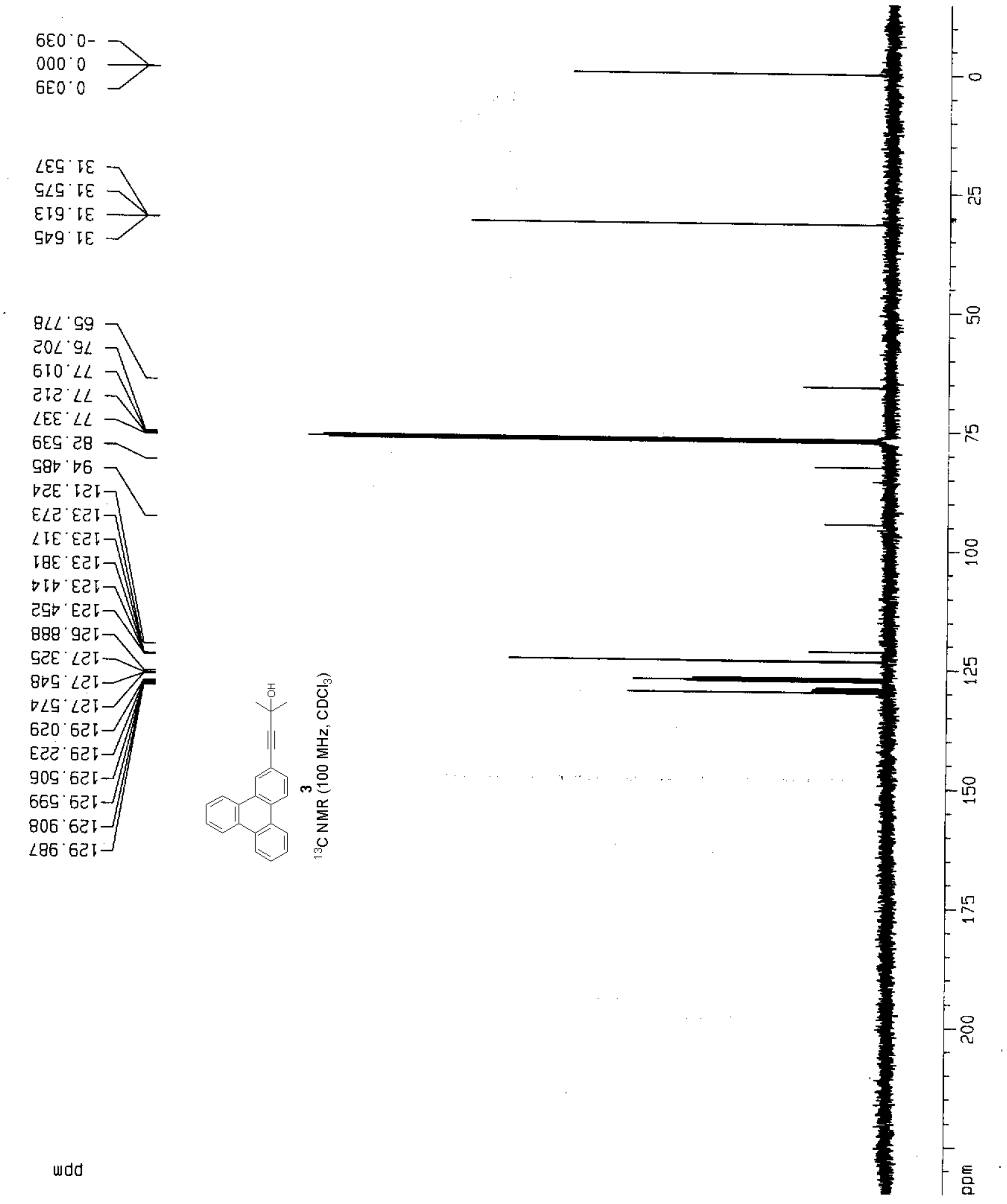

Figure 30: ^{13}C NMR spectrum of **3** in $CDCl_3$.

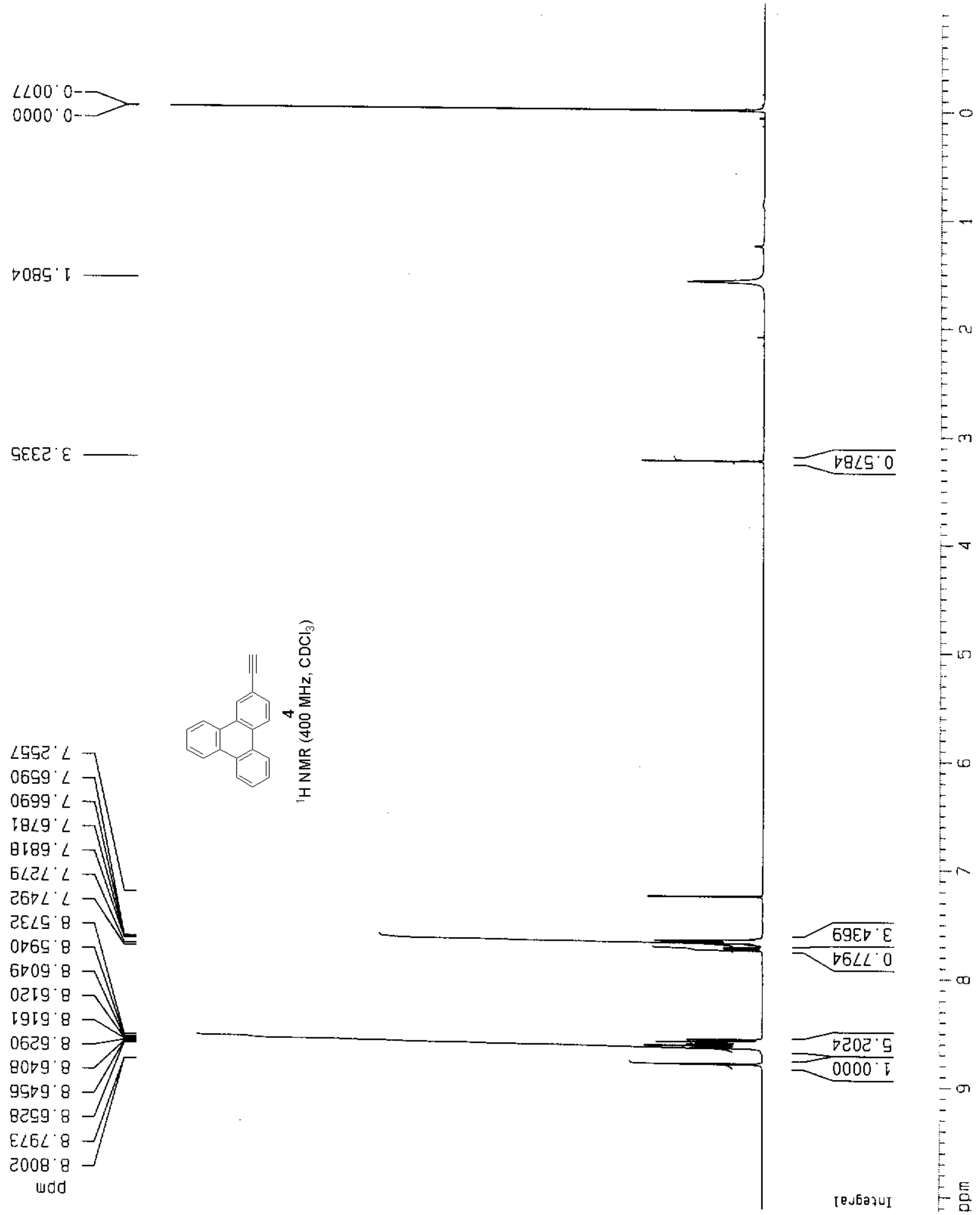

Figure 31: ^{1}H NMR spectrum of **4** in $CDCl_3$.

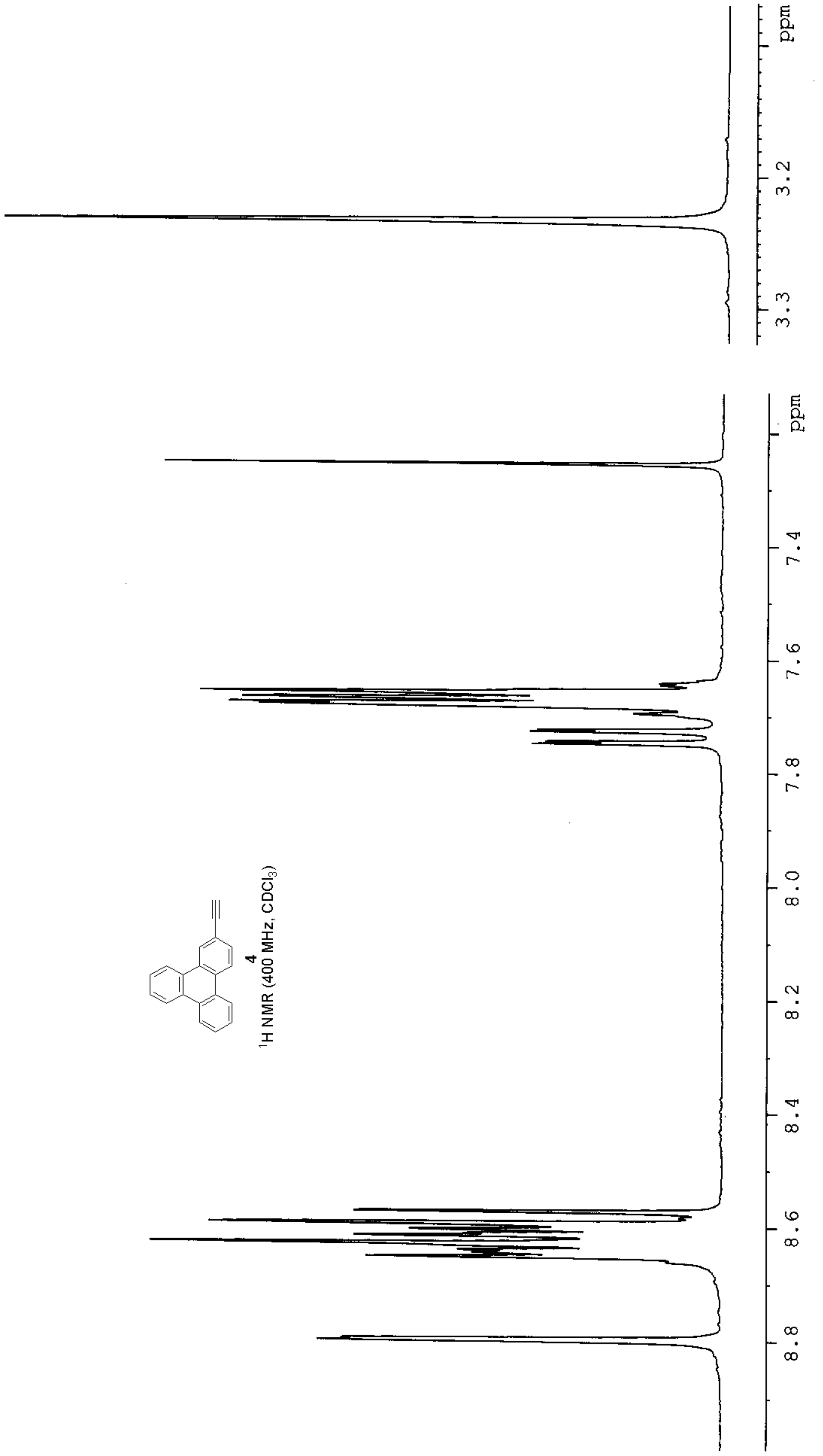

Figure 32: Expanded aromatic region of ^{1}H NMR spectrum of **4** in $CDCl_3$.

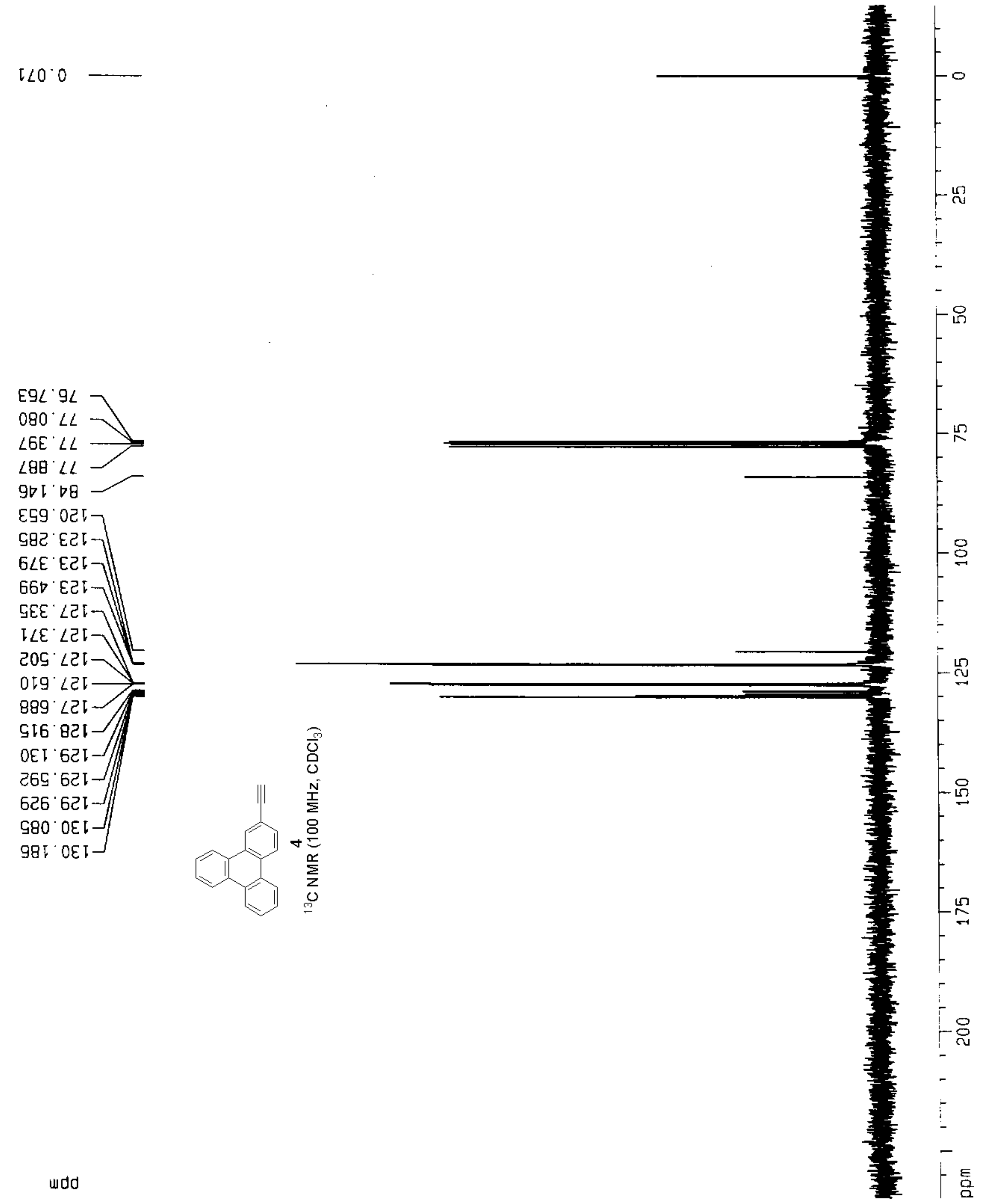

Figure 33: ^{13}C NMR spectrum of **4** in $CDCl_3$.

Calculations were performed using PC Spartan Pro Version 1.1 program (Wavefunction, Inc., Irvine, CA). DFT method using SVWN/DN* basis set was used to obtain the optimized geometries and HOMO-LUMO surfaces.

Coordinates for Compound **1c**.

		Atom	Cartesian Coordinates (Angstroms) X	Y	Z
1	H	H1	-3.6968730	0.0000000	3.4443294
2	C	C1	-4.3370015	0.0000000	2.5578499
3	C	C2	-5.9995252	0.0000000	0.3650627
4	C	C3	-3.7613125	0.0000000	1.2793262
5	C	C4	-5.6994214	0.0000000	2.7384899
6	C	C5	-6.5417825	0.0000000	1.6296128
7	C	C6	-4.6149601	0.0000000	0.1546225
8	H	H3	-6.1181602	0.0000000	3.7508865
9	H	H4	-7.6296020	0.0000000	1.7596379
10	H	H6	-6.6778072	0.0000000	-0.4926710
11	C	C7	-2.3296664	0.0000000	1.0960825
12	C	C8	0.4680835	0.0000000	0.7431860
13	C	C9	-1.4505403	0.0000000	2.1893029
14	C	C10	-1.7793795	0.0000000	-0.2055253
15	C	C11	-0.3915774	0.0000000	-0.3482540
16	C	C12	-0.0902785	0.0000000	2.0331289
17	H	H8	-1.8491105	0.0000000	3.2072697
18	H	H10	0.0581810	0.0000000	-1.3445957
19	H	H11	0.5722642	0.0000000	2.9050023
20	C	C13	-2.6550366	0.0000000	-1.3622005
21	C	C14	-4.3524055	0.0000000	-3.5907164
22	C	C15	-2.1451252	0.0000000	-2.6677375
23	C	C16	-4.0535178	0.0000000	-1.1825748
24	C	C17	-4.8750467	0.0000000	-2.3186647
25	C	C18	-2.9725136	0.0000000	-3.7673274
26	H	H7	-1.0636084	0.0000000	-2.8299058
27	H	H14	-5.9620151	0.0000000	-2.2005742
28	H	H15	-2.5431091	0.0000000	-4.7753403
29	H	H16	-5.0217535	0.0000000	-4.4581155
30	C	C19	1.8605023	0.0000000	0.5717179
31	C	C20	3.0737131	0.0000000	0.4452907
32	C	C21	4.4631404	0.0000000	0.2731857
33	C	C22	7.2374154	0.0000000	-0.0938720
34	C	C23	5.3322690	0.0000000	1.3750399
35	C	C24	5.0156058	0.0000000	-1.0157054

36	C	C25	6.3802376	0.0000000	-1.1983663
37	C	C26	6.6982549	0.0000000	1.1953991
38	H	H9	4.9095414	0.0000000	2.3856026
39	H	H12	4.3449124	0.0000000	-1.8816960
40	H	H13	6.8048195	0.0000000	-2.2077497
41	H	H17	7.3695153	0.0000000	2.0608235
42	C	C27	8.6403112	0.0000000	-0.2836983
43	N	N1	9.7920106	0.0000000	-0.4541113

E(DFT) = -1083.12216 VWN
-1092.93870 pBP86

Coordinates for Compound **1g**.

Atom			Cartesian Coordinates (Angstroms) X	Y	Z
1	H	H1	-4.0968524	-3.5003312	-0.0173063
2	C	C1	-4.7886074	-2.6421025	-0.0165897
3	C	C2	-6.5517087	-0.5335511	-0.0363184
4	C	C3	-4.2773144	-1.3226051	-0.0004412
5	C	C4	-6.1359417	-2.8866948	-0.0288369
6	C	C5	-7.0314039	-1.8287022	-0.0442231
7	C	C6	-5.1832613	-0.2615616	-0.0194876
8	H	H3	-6.5030787	-3.9141975	-0.0292709
9	H	H4	-8.1148083	-2.0046437	-0.0546747
10	H	H6	-7.2761685	0.2873268	-0.0450399
11	C	C7	-2.8504586	-1.0534600	0.0250918
12	C	C8	-0.0729650	-0.5437950	0.0724385
13	C	C9	-1.9195889	-2.0961624	0.0424448
14	C	C10	-2.3623991	0.2639521	0.0243434
15	C	C11	-0.9879239	0.4966258	0.0564116
16	C	C12	-0.5731798	-1.8682234	0.0606397
17	H	H8	-2.2702864	-3.1333186	0.0397025
18	H	H10	-0.5833842	1.5161984	0.0681035
19	H	H11	0.1346563	-2.7028803	0.0708879
20	C	C13	-3.3000320	1.3659595	0.0001811
21	C	C14	-5.1148549	3.5112811	-0.0200283
22	C	C15	-2.8611497	2.6952698	-0.0163989
23	C	C16	-4.6906074	1.1114368	-0.0112162
24	C	C17	-5.5697963	2.2058571	-0.0197166
25	C	C18	-3.7412892	3.7474494	-0.0283724
26	H	H7	-1.7941232	2.9203999	-0.0188102
27	H	H14	-6.6499838	2.0373035	-0.0221044
28	H	H15	-3.3632164	4.7639481	-0.0477067

29	H	H16	-5.8262750	4.3459722	-0.0265238
30	C	C19	1.3231596	-0.3274806	0.0761250
31	C	C20	2.5413729	-0.2077439	0.0663680
32	C	C21	3.9465173	-0.0791257	0.0375135
33	C	C22	6.7743391	0.1041982	-0.0187506
34	C	C23	4.6948497	0.0455062	1.2150280
35	C	C24	4.6527177	-0.1153753	-1.1568563
36	C	C25	6.0237921	-0.0297969	-1.1918528
37	C	C26	6.0630003	0.1199951	1.2078670
38	H	H9	4.1663840	0.0564479	2.1855872
39	H	H12	4.1030692	-0.2195991	-2.0934493
40	H	H13	6.5297470	-0.0707051	-2.1649206
41	H	H17	6.6024600	0.2074788	2.1438841
42	N	N1	8.1424714	0.1903733	-0.0585679
43	C	C27	8.8794467	-0.0171439	1.1594516
44	H	H19	8.6134562	0.7287200	1.9264365
45	H	H20	8.7189437	-1.0295695	1.5908939
46	H	H21	9.9490638	0.1050378	0.9608313
47	C	C28	8.8166958	-0.0897427	-1.2963487
48	H	H18	9.8984434	0.0601083	-1.1635834
49	H	H22	8.6494460	-1.1476612	-1.6457306
50	H	H23	8.4840579	0.5902048	-2.0941324

E(DFT) = -1124.44462 VWN
-1134.66136 pBP86

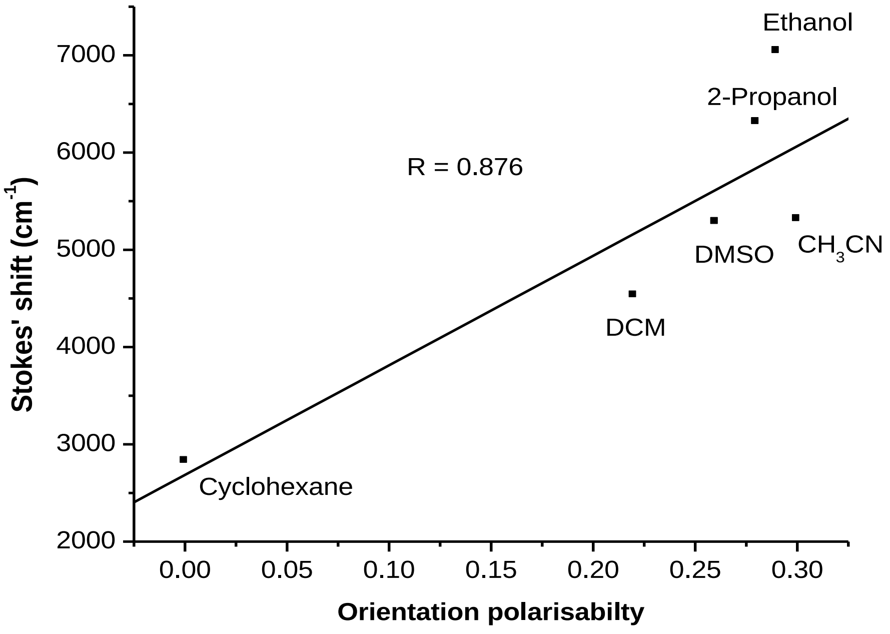

Figure 34: Lippert-Mataga plot for **1d**.

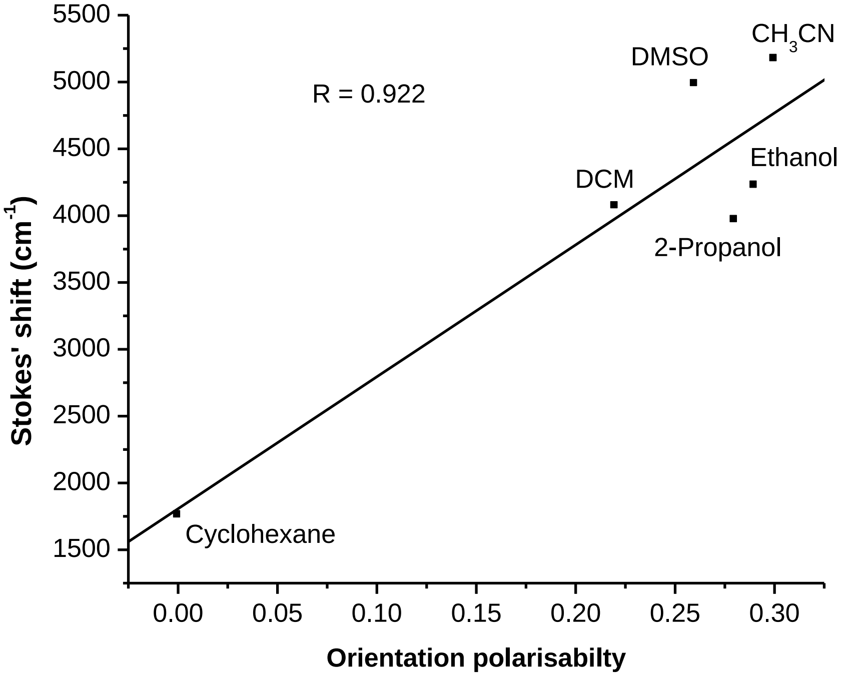

Figure 35: Lippert–Mataga plot for **1f**.

References

1. Lakowicz J. R.; *Principles of Fluorescence Spectroscopy,* 2nd ed.; Kulwer Academic: New York, 1999; pp 52–54.
